# A wound-friendly antibacterial hyaluronic acid dressing with on-demand removability for infected wound healing

**DOI:** 10.1186/s40824-023-00340-7

**Published:** 2023-05-01

**Authors:** Datao Hu, Jinpeng Wen, Xinxin Zhao, Kailai Liu, Yuchen Zhang, Yizhuo Bu, Ke Wang

**Affiliations:** 1 School of Pharmacy, Health Science Center, Xi’an Jiaotong University, Xi’an, 710061 China; 2 State Key Laboratory of Natural and Biomimetic Drugs, Peking University, Beijing, China

**Keywords:** On-demand removal, Injectable, Antibacterial, Wound dressing

## Abstract

**Background:**

Antibacterial activity and on-demand removability are key characteristics governing the effectiveness of clinic wound dressing. However, the excellent tissue adhesion of new dressings is often overemphasized without a detailed discussion of dressing replacement. Besides, the inherent antibacterial ability of dressings is beneficial for promoting the healing of infected wound. Therefore, we rationally design an injectable antibacterial wound dressing with on-demand removability to accelerate infected wound healing.

**Method:**

We design this wound dressing with a simple and feasible method based on the electrostatic self-assembly of hyaluronic acid and ε-polylysine. We investigated the efficacy of this dressing in terms of its microtopography, rheology, self-healing performance, adhesive ability, antimicrobial, hemostatic, on-demand removal properties, and wound healing promotion through various tests.

**Results:**

The prepared dressing possesses injectability, self-healing ability and antibacterial activity, showing NaCl-triggered on-demand dissolution due to the disruption of electrostatic interactions. When used as dressings for healing full-thickness wounds, it could effectively accelerate wound healing by killing bacteria, downregulating inflammation, promoting collagen deposition, enhancing keratinocyte migration and angiogenesis due to its excellent adhesion ability, favorable hemostatic property, and potent antibacterial performance.

**Conclusion:**

All results indicate that this is a simple and practical dressing for clinical application. This strategy provides a novel idea for developing on-demand removal dressings with antibacterial and injectable properties.

**Supplementary Information:**

The online version contains supplementary material available at 10.1186/s40824-023-00340-7.

## Background

The integrity of skin tissue is the premise for its function [1, 2]. Nevertheless, the skin is highly vulnerable to the external environment in daily life [3]. It has been reported that millions of people suffer from skin injury for various factors every year, and in serious cases, it can cause disability or even death [4]. Wound healing is generally believed to be a complex and precise process that starts immediately after wound formation [5]. The four sub-stages of wound healing (hemostasis, inflammation, proliferation, and tissue remodeling) influence each other and are highly coordinated [6, 7]. Wounds can be divided into superficial wounds and full-thickness wounds according to the depth. However, regardless of the wound type, it is necessary to deal with wounds properly.

Skin injury is a common clinical problem, and covering wounds with medical dressings is a common aid for wound care [8, 9]. Additionally, infections caused by bacterial contamination are one of the key factors leading to delayed wound healing [10], with the majority of skin and soft tissue infections resulting from *Staphylococcus aureus* (*S. aureus*) [11, 12]. More seriously, more than 30% of infections still develop chronic wounds or recur within 3 months of appropriate antimicrobial treatment [13]. To avoid wound infections, dressings should have good antibacterial activity [7, 14, 15]. Dressings with inherent antibacterial activity generally have longer antimicrobial activity and better safety than dressings that release antimicrobial agents [16, 17]. Therefore, the inherent antibacterial property of dressings is of great concern.

Moreover, the adhesion between wounds and dressings is another significant challenge in applications [18–20]. According to Hollinworth et al., when changing dressings, care should be taken to prevent wound injury and avoid causing pain to patients. Notably, 81% of the patients reported experiencing the most pain when dressing changes [21]. Generally, skin injury is often accompanied by the wound exudate that, when dry, infiltrates the structure of the dressings so that dressings will adhere to the wound. Alternatively, proteins in the wound exudate may form hydrogen bonds with the dressings, thereby leading to adhesion [22]. Traditional dressings tend to adhere to wounds, resulting in secondary trauma during dressing changes [23, 24]. Disappointingly, the excellent tissue adhesion of new dressings is often overemphasized, with no detailed discussion of the on-demand removal property of the dressings. At present, several on-demand removal dressings have been developed [25]. These dressings have good adhesion to the tissue and wound. When dressings need to be replaced, their sensitivity to external stimulation can be used to facilitate removal. However, most of these on-demand removal dressings are sensitive to acid, alkali, or special substances [26–28], which will influence the wound environment, even causing secondary injury. Therefore, developing mild as well as on-demand removal dressings is necessary to prevent wound adhesion.

**Scheme 1:**
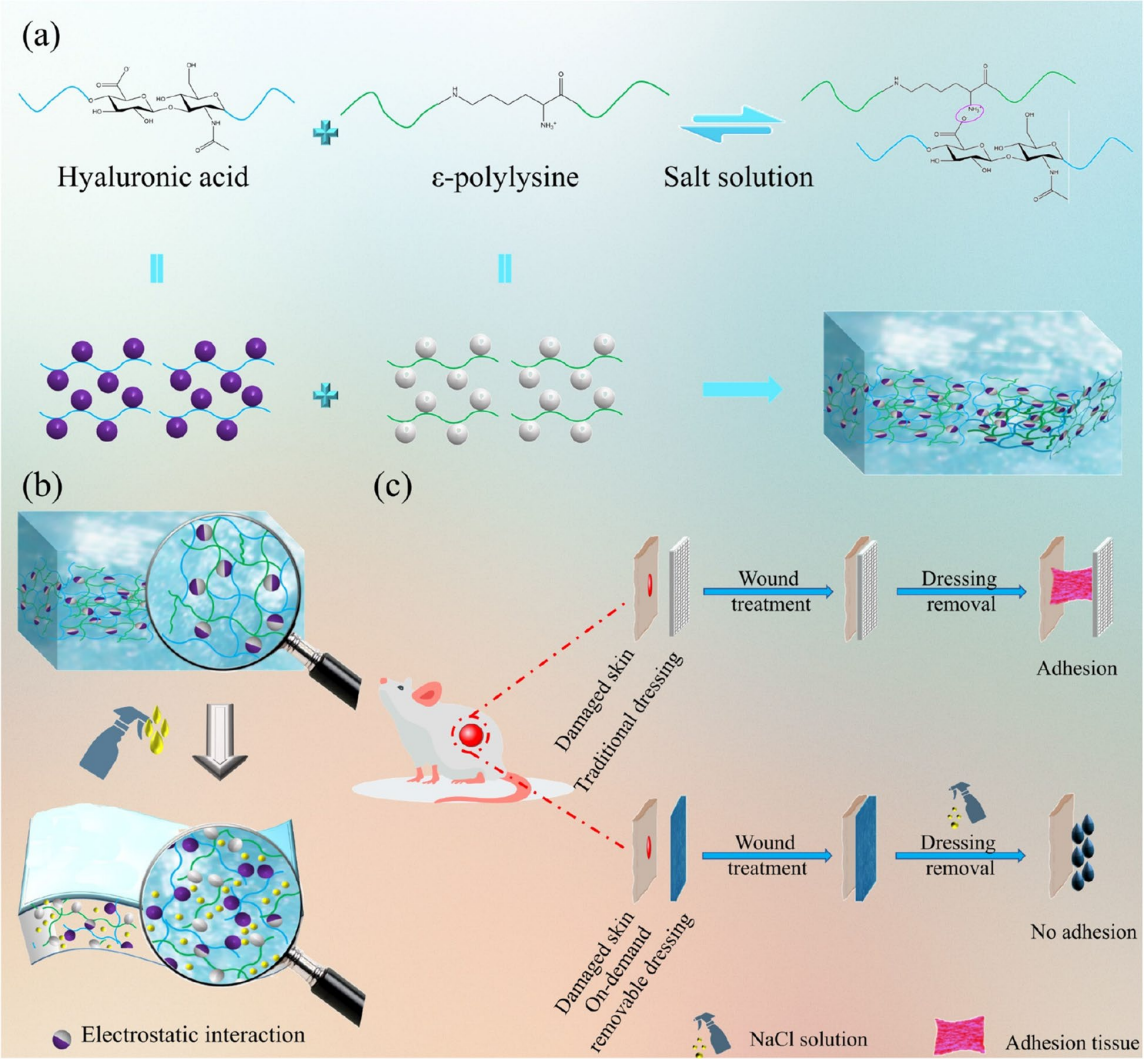
Schematic illustration of the fabrication of injectable and on-demand removal dressing. (**a**) HA-EPL coacervates were prepared based on the reaction of HA and EPL. (**b**) HA-EPL coacervates could be dissolved when exposed to NaCl solution owing to the destruction of electrostatic interactions. (**c**) HA-EPL coacervates enabled the rapid recovery of open wounds and could be dissolved on demand through NaCl treatment

Until now, extensive efforts have been devoted to designing new dressings with desired therapeutic efficacy, such as films, hydrocolloids, foams, sponges, and hydrogels, under the guidance of the moist healing concept [29]. Nevertheless, complex design routes, using of organic solvents and the residues of cross-linking agents or initiators have greatly hindered the additional applications of these new dressings [30]. Inspired by the above views, we intended to prepare a new type of injectable, antibacterial and on-demand removal dressing (HA-EPL) with a simple and feasible method (Scheme 1). It is an emerging class of injectable wound dressings formed with hyaluronic acid (HA) and ε-polylysine (EPL) through electrostatic self-assembly. Interestingly, HA-EPL is injectable, does not require complex synthesis or modification, and simultaneously performs many favorable functions as a wound dressing. Dynamic cross-linking endows HA-EPL with preferable injectable and self-healing properties via electrostatic interactions. Besides, the inherent antibacterial activity of HA-EPL enabled it to effectively kill bacteria in infected wounds. More importantly, HA-EPL not only has good tissue adhesion but is also sensitive to salt solutions, implying that it can be removed as required to achieve painless dressing change, thus reducing damage to new tissues and improving patient compliance. Taken together, all of these desirable properties make HA-EPL an ideal dressing for promoting the infected wound healing and tissue regeneration. This method also provides an important idea for developing injectable, on-demand removal antibacterial dressings.

## Experimental section

### Materials

Hyaluronic acid sodium salt (MW = 150,000 ~ 250,000) was provided by Shanghai yuanye Bio-Technology Co., Ltd. (Shanghai, China). ε- Poly-L-lysine was obtained from Zhengzhou Bainafo Bioengineering Co., Ltd. (Zhengzhou, China). Fibroblast cells (L929) were purchased from the American Type Culture Collection (ATCC, VA, USA).

### Preparation of HA-EPL coacervates

4% of HA and 4, 6 and 8% EPL solutions (w/v) were prepared, respectively. Then, the prepared HA solution and EPL solution were fully oscillated and mixed at a volume ratio of 10:3. The molar ratios of carboxyl groups to amino groups are 1: 0.66, 1: 1 and 1: 3, respectively. After standing for 10 min, HA-EPL coacervates could be observed. To observe the coalescence process of HA-EPL coacervate droplets under a microscope, HA solution (10 μL) and 8% EPL (3 μL) solution were dropped on the glass slides and then covered with a coverslip to prepare the sample. HA-EPL coacervates with EPL concentrations of 4, 6, and 8% were named as 4% HA-EPL, 6% HA-EPL, and 8% HA-EPL.

### Characterization of HA-EPL coacervates

HA-EPL coacervates were characterized by the Fourier transform infrared spectrometer (FT-IR), X-ray diffraction (XRD) and scanning electron microscopy (SEM), respectively. The details are shown in the Methods section in the Supporting Information.

### Rheological tests

The rheological tests of HA-EPL coacervates were performed using an Anton-Paar rheometer (MCR302, Austria) with 20 mm flat plates and a 1.0 mm gap at 37 °C. Stress sweep tests, frequency sweep tests, shear viscosity tests and step-strain-sweep tests were performed under suitable conditions, respectively. The details are shown in the Methods section in the Supporting Information.

### Self-healing and adhesive properties

The self-healing ability of 8% HA-EPL was first evaluated macroscopically. Then, the tissue adhesion property of 8% HA-EPL was assessed using porcine skin and the main organs of mice. The details are shown in the Methods section in the Supporting Information.

### The dissolution behavior of HA-EPL coacervates

The dissolution behavior of HA-EPL coacervates was first evaluated macroscopically. Then, the dissolution behaviors of 4, 6 and 8% HA-EPL were also analyzed by the rheological tests and SEM. The details are shown in the Methods section in the Supporting Information.

### In vitro antibacterial activity evaluation

The surface antibacterial activities of HA-EPL coacervates for *S. aureus* (ATCC 12228) and *E. coli* (ATCC 8739) were evaluated by the colony count method. The details are shown in the Methods section in the Supporting Information.

### In vitro cytotoxicity of HA-EPL coacervates

The cytotoxicity of HA-EPL coacervates to L929 cells was detected by MTT assay. The details are shown in the Methods section in the Supporting Information.

### In vitro and in vivo hemostasis ability

The coagulation efficacy of samples was evaluated via a tube reversion test. Then, the mouse-tail amputation model and acute liver hemostasis model were performed to further evaluate the hemostasis ability of HA-EPL coacervates. The details are shown in the Methods section in the Supporting Information.

### In vivo healing of full-thickness wounds

The therapeutic effect of 8% HA-EPL was evaluated using a mouse full-thickness skin defect model. The details are shown in the Methods section in the Supporting Information.

### In vivo healing of full-thickness infected wounds

Three 13 mm diameter full-thickness round defects were created in the center of the shaved back of SD rats (male, 250 ~ 280 g). Then, 80 μL of *S. aureus* (10^8^ CFU mL^− 1^) was added to the wound to form the infection, and the dressing was covered on the wound after 1 h. The details are shown in the Methods section in the Supporting Information.

### Histological evaluation and immunofluorescent staining

To further investigate the mechanism of 8% HA-EPL on wound healing, the wound tissues were collected for histological evaluation and immunofluorescent staining. The details are shown in the Methods section in the Supporting Information.

### Quantitative real-time PCR

The wound tissues of infected rats on day 10 were collected for the quantitative real-time PCR. The details are shown in the Methods section in the Supporting Information.

### Statistical analysis

All results are expressed as mean ± SD. GraphPadPrism7.0 (LaJolla, CA, USA) software was used for statistical analysis. The data were analyzed using one-way ANOVA followed by Tukey’s test, where values of *P < 0.05, **P < 0.01, and ***P < 0.001 were considered statistically significant.

## Results and discussion

### Preparation, characterizations and rheology properties of HA-EPL Coacervates

**Fig. 1:**
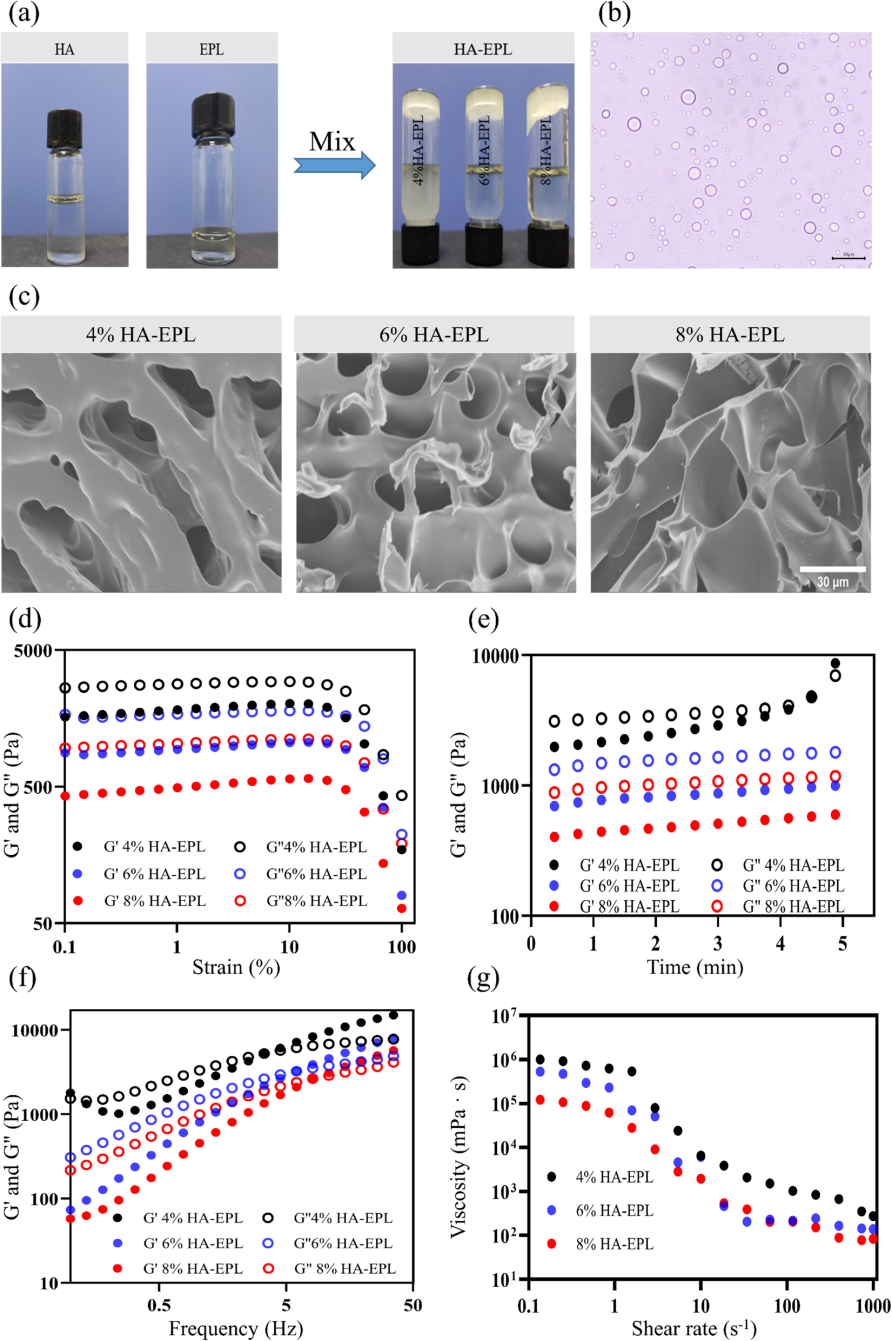
Characterizations of prepared HA-EPL coacervates. **a** The synthetic process of HA-EPL coacervates. **b** Distribution of newly formed HA-EPL droplets observed under a microscope. **c** SEM images of HA-EPL coacervates. **d**-**g** Rheological properties of HA-EPL coacervates. **d** Strain sweep. **e** Time sweep. **f** Frequency sweep. **g** Shear rate sweep

With a simple and feasible method, a new type of injectable, self-healing, and on-demand removal dressing was successfully prepared. As presented in Fig. 1a, after shaking and standing for 10 min, the mixture of HA and EPL solutions was completely separated into a light yellow coacervate phase and a corresponding supernatant. It can be observed that the coacervate droplets with sizes of several micrometers to tens of micrometers were dispersed in the aqueous environment (Fig. 1b), where large droplets consisted of small droplets [31]. SEM images of HA-EPL coacervates were shown in Fig. 1c. The pore diameter of 6% HA-EPL was close to that of 8% HA-EPL, while 4% HA-EPL showed a larger pore diameter and more extended size. This might be attributed to the insufficient number of amino groups and the weak electrostatic interactions in 4% HA-EPL [32]. Nevertheless, the presentation of the porous structure suggested that HA-EPL coacervates possessed the potential to be used as a dressing.

Electrostatic interactions and hydrogen bonds synergistically facilitated the formation of HA-EPL coacervates by FTIR analysis (Fig. S1). In the spectrum of 8% HA-EPL (HA-EPL coacervates with EPL concentrations of 4, 6, and 8% were named as 4% HA-EPL, 6% HA-EPL, and 8% HA-EPL.), peaks at 1413 cm^− 1^ and 1618 cm^− 1^ which were related to the stretching vibration of the carboxylic group weakened or disappeared because HA was involved in the formation of HA-EPL coacervates. Besides, a decrease in the intensity of bands associated with amide I, II, and III (1674 cm^− 1^, 1564 cm^− 1^, and 1255 cm^− 1^) was also observed. This change might be attributed to electrostatic interactions between the amino groups of EPL and the carboxyl groups of HA during the formation of HA-EPL coacervates [32]. In addition to electrostatic interactions, the O-H stretching band in HA (3405 cm^− 1^) and the N-H stretching band in EPL (3419 cm^− 1^) were shifted to 3431 cm^− 1^, meaning that hydrogen bonds formed in the formation process of HA-EPL coacervates [33]. Furthermore, XRD spectra showed that the structure of HA and EPL changed during the coacervation process, which may be caused by physical interactions between protein and polysaccharide (Fig. S2).

The viscoelastic behaviors of HA-EPL coacervates were determined to further investigate their mechanical properties. In strain sweep tests (Fig. 1d), the values of *G’* and *G“* for HA-EPL coacervates prepared by different ratios were obtained. HA-EPL coacervates exhibited a linear viscoelastic region and values of *G”* > *G’* when the strain ranged from 0.1 to 10%. In the time sweep tests (Fig. 1e), the values of *G“* were always higher than those of *G’* for 6% and 8% HA-EPL, and no intersection was observed during the test time (0–5 min). However, for 4% HA-EPL, the *G’* and *G”* values marginally increased and finally intersected over time, thus indicating that the network structure of 4% HA-EPL was disturbed and better stability was achieved with 6 and 8% HA-EPL. In the frequency sweep tests (Fig. 1f), *G’* and *G“* values for 4%, 6% and 8% HA-EPL gradually increased within the test frequency of 0.1-35 Hz. Besides, *G”* > *G’* of 4, 6, and 8% HA-EPL were observed when frequencies were 4.5, 6.0, and 10.8 Hz, respectively. This further indicated that the stability of 8% HA-EPL was better than those of 4 and 6% HA-EPL. Besides, dressings with the injectable property have various advantages such as ease of operation and matching with the wound perfectly (Fig. S3) [34]. As shown in Fig. 1g, the viscosity of 4, 6 and 8% HA-EPL significantly decreased with the increase of shear rate, suggesting that HA-EPL coacervates exhibited a typical shear-thinning behavior and the preferable injectability. Also, HA-EPL coacervates could be continuously extruded from the needle (Movie S1) and draw letters of “XJTU” without clogging (Fig. S4). The above results demonstrated that 8% HA-EPL with the preferable injectability, which could maintain a stable state for a long time, possesses potential as a medical dressing.

### Self-healing, tissue-adhesive and on-demand removal properties of HA-EPL Coacervates

**Fig. 2:**
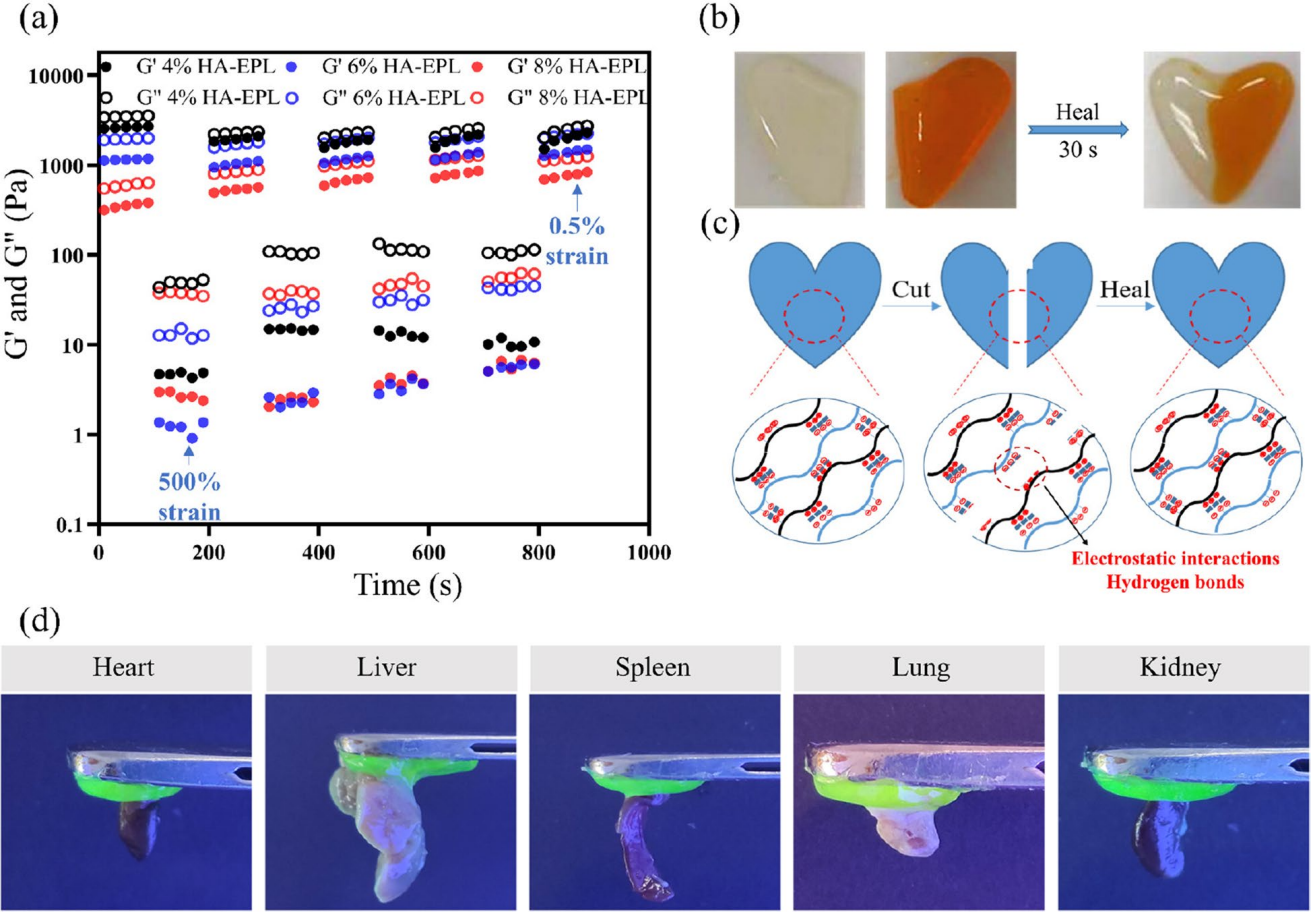
Self-healing and tissue-adhesive properties of HA-EPL coacervates. **a** Step strain-sweep test. **b** The macroscopic self-healing ability of 8% HA-EPL. **c** Schematic illustration of the healing mechanism for HA-EPL coacervates. **d** Adhesion of 8% HA-EPL to various organs of mice

The excellent self-healing ability enabled HA-EPL coacervates to cope with various accidental dressing damages. Thus, the self-healing ability of HA-EPL coacervates was determined by the step-strain-sweep scanning test. The *G’* and *G”* values of 4, 6, and 8% HA-EPL returned to their original levels when the strain was converted between 0.5 and 500% (Fig. 2a), indicating that the collapsed structure of HA-EPL coacervates could rapidly recover. Besides, this collapse-recovery process could be repeated at least four times, demonstrating that HA-EPL coacervates possess efficient and rapid self-healing ability. Macroscopically, two separate heart-shaped 8% HA-EPL patches healed rapidly in 30 s with almost no cut section, further confirming the excellent self-healing ability of HA-EPL coacervates (Fig. 2b). The self-healing mechanism of HA-EPL coacervates stems from physical dynamic interactions (Fig. 2c). Both electrostatic interactions and hydrogen bonds contribute to the recovery of HA-EPL coacervates.

Good adhesion ensures that the dressing is stably anchored to the skin to protect the wound. As shown in Fig. S5, an 8% HA-EPL patch adhered firmly to the surface of porcine skin and even stuck two separate pieces of porcine skin together very well. in addition, the 8% HA-EPL patch exhibited satisfactory adhesiveness to mice organs (Fig. 2d). This good adhesion performance was mostly attributed to the residual amino groups and carboxyl groups on the surface of HA-EPL coacervates, contributing to HA-EPL coacervates binding on the skin surface [35].

**Fig. 3:**
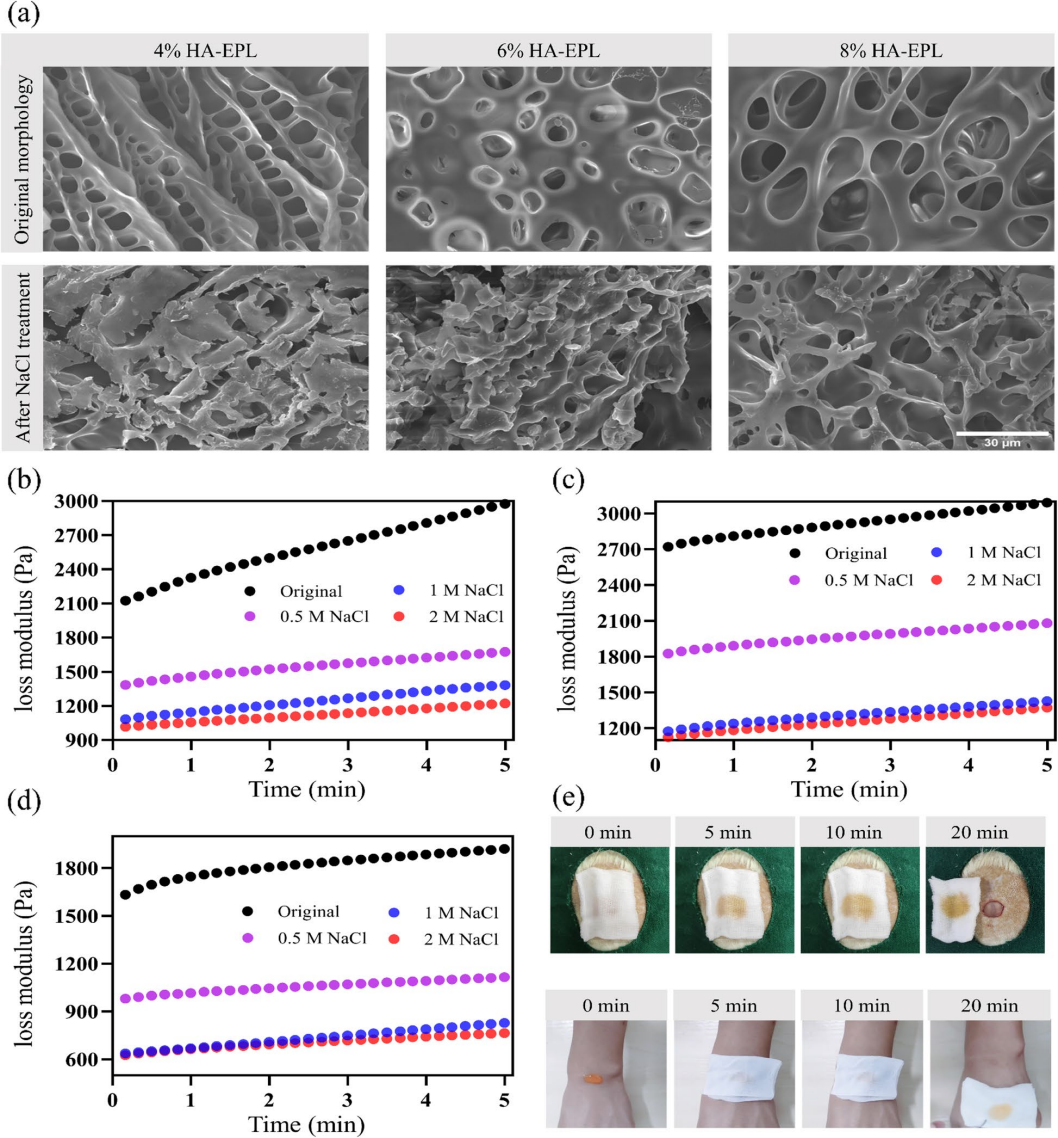
On-demand removal property of HA-EPL coacervates. **a** SEM images of 4, 6 and 8% HA-EPL before and after treatment of 1 M NaCl solution for 20 min. **b** *G“* values of 4% HA-EPL decreased when exposed to 0.5 M, 1 M and 2 M NaCl solutions. **c** *G”* values of 6% HA-EPL decreased when exposed to 0.5 M, 1 M and 2 M NaCl solutions. **d** *G”* values of 8% HA-EPL decreased when exposed to 0.5 M, 1 M and 2 M NaCl solutions. **e** The gauze infiltrated with 1 M NaCl solution could completely remove 8% HA-EPL on the rat skin and human skin

Traditional dressings often cause pain when they were peeled from injured skin. Furthermore, current dressings deliberately emphasize their excellent tissue adhesion, while the replacement of dressings has not been discussed in detail. On-demand removal dressings could reduce patients’ distress due to adjustable adhesion. Consequently, given the ability of the salt solution to weaken the electrostatic interactions between HA and EPL, HA-EPL coacervates were predicted to be sensitive to NaCl solution. As expected, 4, 6, and 8% HA-EPL partially dissolved within 30 min when immersed in different NaCl solution concentrations. Subsequently, 4, 6, and 8% HA-EPL were no longer observed after soaking in NaCl solution for 2 h (Fig. S6). SEM images revealed that 4, 6, and 8% HA-EPL displayed a porous structure, while this porous morphology became irregular after 1 M NaCl solution treatment, indicative of the NaCl solution-induced destruction of HA-EPL coacervates network (Fig. 3a). Furthermore, rheological tests were performed to investigate the dissolution behavior of HA-EPL coacervates. As revealed in Fig. 3b-d, when exposed to 0.5 M NaCl solution for 20 min, the *G“* values of the 4%, 6%, and 8% HA-EPL showed a modest decrease. Following on, the *G”* values of the 4, 6 and 8% HA-EPL showed a substantial decrease if 4, 6 and 8% HA-EPL were immersed in 1 and 2 M NaCl solutions, respectively. Predictably, HA-EPL coacervates could be effectively removed when adhering to the wound. As presented in Fig. 3e, 8% HA-EPL was injected onto a rat wound, after which the gauze, infiltrated with 1 M NaCl solution, was gently placed on the 8% HA-EPL. After 20 min incubation, nearly all of the 8% HA-EPL covered by the NaCl-soaked gauze dissolved (Movie S2). Besides, the process of on-demand removal on the human hand was shown in Movie S3.

### Antibacterial and hemostatic abilities of HA-EPL Coacervates

**Fig. 4:**
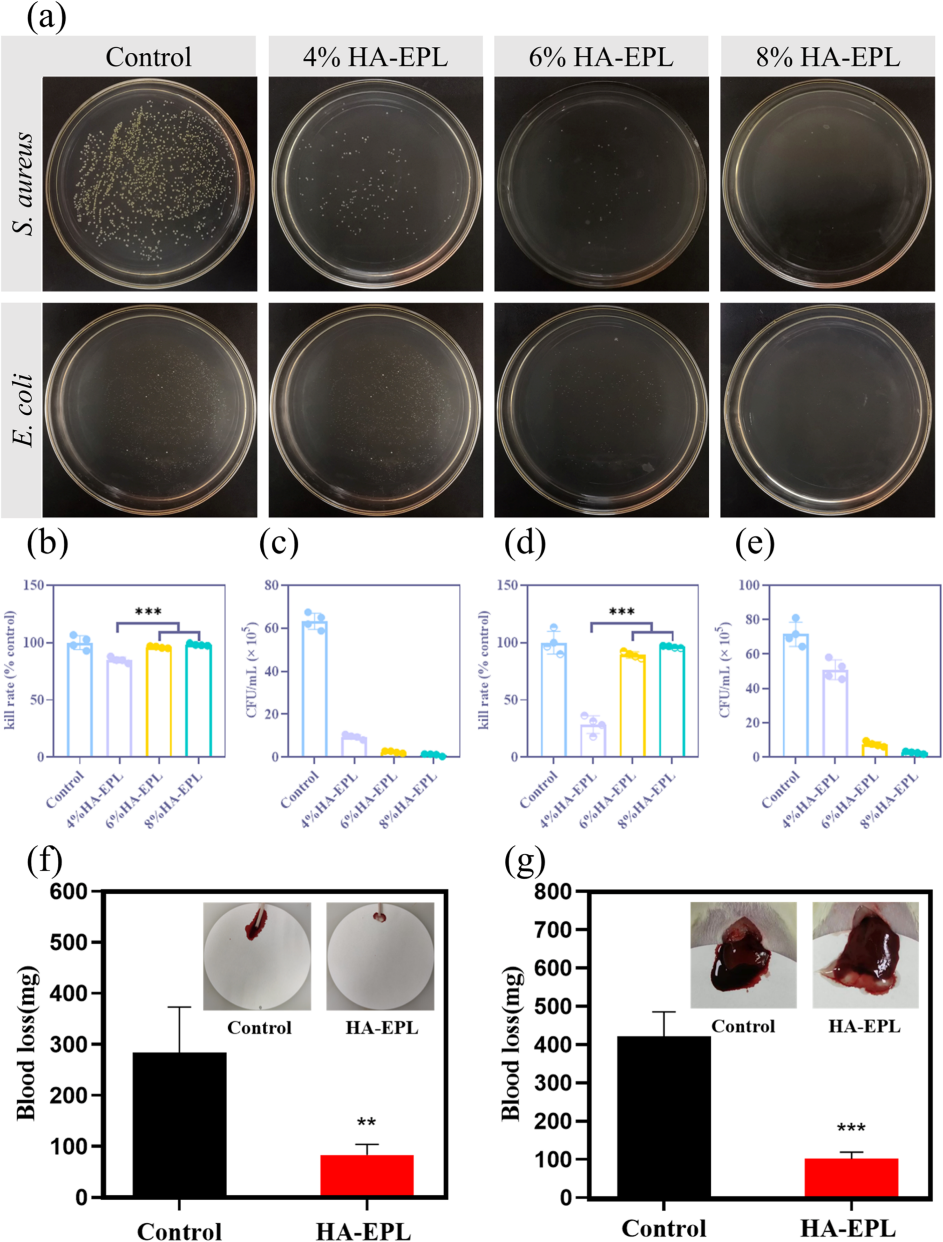
Antibacterial and hemostatic properties of HA-EPL coacervates. **a** Reproduction of *S. aureus* and *E. coli* on Agar plate after contact with HA-EPL coacervates. **b** and **c** Corresponding statistical data of *S. aureus* (*n* = 4). d and e Corresponding statistical data of *E. coli* (*n* = 4). **f** The hemostasis experiment of mouse tail amputation model. **g** The hemostatic experiment of acute hepatic hemorrhage in rats (*n* = 3). *P < 0.05, **P < 0.01, ***P < 0.001

Bacterial infections will delay the wound healing process, so an ideal wound dressing should possess excellent antibacterial activity [36]. In this case, the antibacterial activity of HA-EPL coacervates against *S. aureus* (Gram-positive bacterium) and *E. coli* (Gram-negative bacterium) was evaluated via surface antibacterial activity tests. Following incubation for 1 h, more than 85% of *S. aureus* was killed by 4, 6, and 8% HA-EPL (Fig. 4a-c), thus demonstrating the excellent antibacterial ability of HA-EPL coacervates against *S. aureus*. Notably, only 6 and 8% HA-EPL exhibited outstanding bacteriostatic effects on *E. coli*, whereas 4% HA-EPL showed poor bacteriostatic effects (Fig. 4a, d and e). In general, because of the differences in the cell wall and cell membrane structures between Gram-negative and Gram-positive bacteria, EPL was more sensitive to Gram-positive bacteria, meaning that HA-EPL coacervates had a better killing effect on *S. aureus* [37, 38]. Furthermore, the bacteriostatic effect was enhanced by increasing the relative content of EPL in HA-EPL coacervates. After examining previous experimental results, 8% HA-EPL was chosen for subsequent experiments owing to its better antibacterial ability and excellent mechanical properties.

The coagulation efficacy of samples was evaluated via a tube reversion test. As displayed in Fig. S7, the precursor solution of 8% HA-EPL could not coagulate heparinized rat whole blood completely, while 8% HA-EPL itself could form a blood-hybrid coacervate. This was because positively charged EPL formed flocs with negatively charged blood cells. Furthermore, the in vivo hemostatic performance of 8% HA-EPL was evaluated by the rat liver bleeding model and the mouse tail-amputation model. For both bleeding models,8% HA-EPL exhibited excellent hemostatic performance. In the mouse tail-amputation model, the blood loss of the 8% HA-EPL group was significantly reduced compared with that of the control group (Fig. 4f). Correspondingly, the quantitative analysis results indicated that the blood loss in the 8% HA-EPL group (83 mg) was remarkably less than that in the control group (284 mg). Besides, 8% HA-EPL exhibited good hemostatic ability in the rat liver bleeding model. As shown in Fig. 4g and Movie S4, 8% HA-EPL immediately adhered to the wound surface and sealed the open blood vessels after liver resection. Only a little bleeding was observed during this process, significantly different from the control group. According to the quantitative analysis, the blood loss was 421 mg in the control group and only 102 mg after treatment with 8% HA-EPL (*P* < 0.001), illustrating the good hemostatic effect of the 8% HA-EPL.

### Toxicity evaluation

The safety of dressings is a prerequisite for application. First, we evaluated the cytotoxicity of HA-EPL coacervates via MTT assay. As depicted in Fig. S8, with increasing concentrations of HA-EPL coacervates, the cell viability was similar, which was higher than 80%. This result showed that HA-EPL coacervates had low cytotoxicity and good biocompatibility. Besides, after treatment with 8% HA-EPL, major organs were collected for organ toxicity evaluation. As illustrated in Fig. S9, there was no significant difference between normal and HA-EPL groups. No obvious tissue degeneration or necrosis was found after treatment with 8% HA-EPL. In conclusion, these results showed that HA-EPL coacervates possessed excellent safety, which could be used as an ideal dressing.

### In vivo full-thickness wound healing

**Fig. 5:**
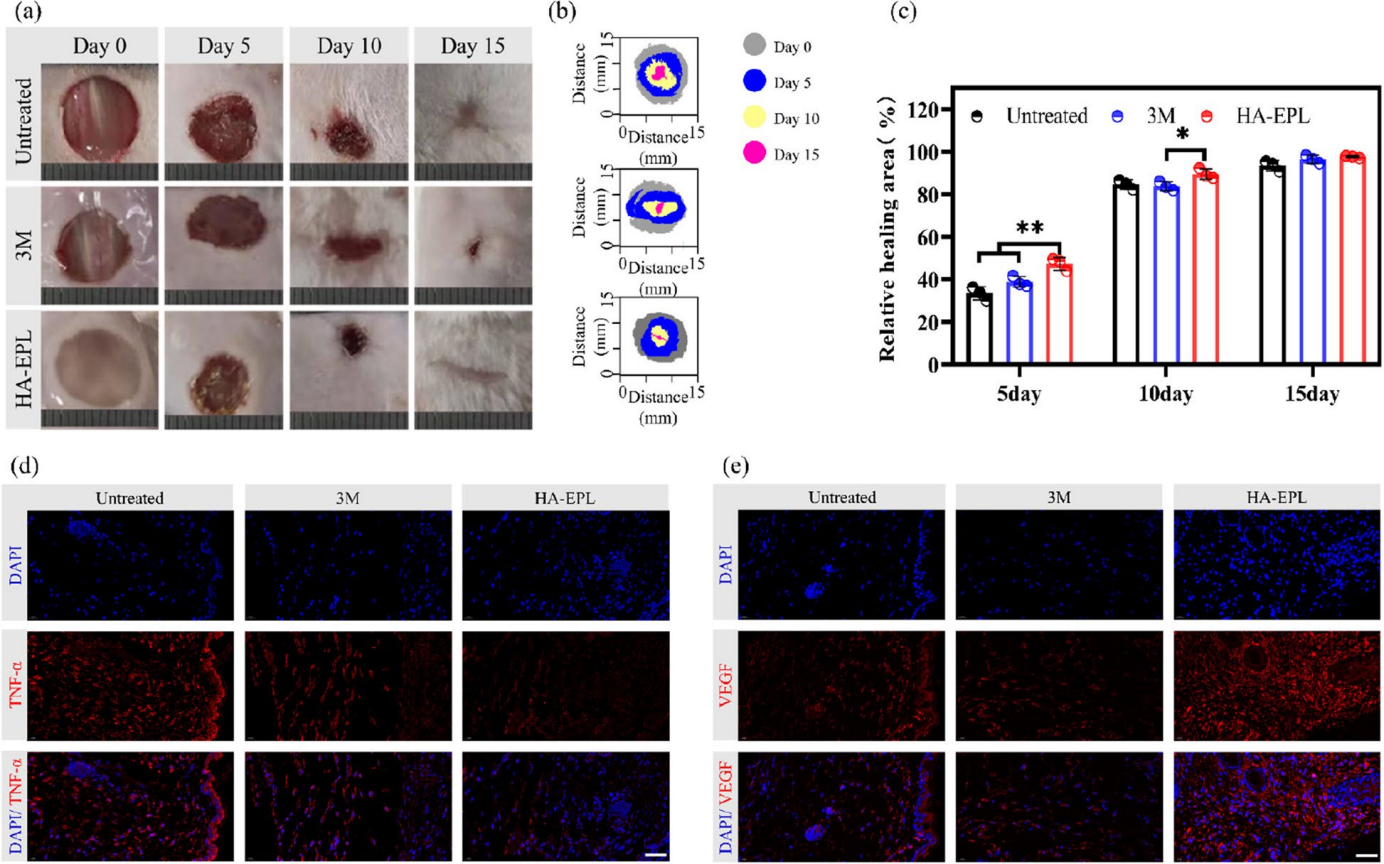
Treatment efficiency of different groups in the full-thickness wound. **a** Representative photographs of wounds with different treatments. **b** Wound healing track in each group. **c** Quantification of wound contraction rate (*n* = 3). **d** Immunofluorescence staining of TNF-α (red) and nuclei (blue) on day 15 in the full-thickness wound. **e** Immunofluorescence staining of VEGF (red) and nuclei (blue) on day 15 in the full-thickness wound. *P < 0.05, **P < 0.01, Scale bar: 50 μm

The above results proved that 8% HA-EPL possessed desirable properties for an ideal wound dressing. Consequently, we evaluated the efficiency of 8% HA-EPL using a mouse full-thickness skin defect model. Dorsal full-thickness skin wounds with a diameter of 10 mm were created and subsequently treated with control (untreated group), Tegaderm film (3 M group), and 8% HA-EPL (HA-EPL group), respectively. Representative images of wounds in each group at predetermined time points are summarized in Fig. 5a. Macroscopically, the therapeutic effect of the HA-EPL group was better than that of the untreated group and 3 M group (Fig. 5a and b). Correspondingly, the wound areas were quantitatively measured at predetermined times (Fig. 5c). On the fifth day, the wound contraction in the HA-EPL group was most pronounced, with a relative healing area of 47.36%, which was higher than that in untreated (33.44%) and 3 M (38.86%) groups. After healing for 10 days, all the wounds exhibited significant contraction, and the wound contraction in the HA-EPL group remained higher than that in the untreated group and the 3 M group (*P* < 0.05). The wounds in the three groups were almost completely repaired after 15 days, but the wounds treated with 8% HA-EPL were still better than those in the other groups. Besides, the results of H&E staining and Masson staining suggested that 8% HA-EPL effectively downregulated inflammation and recovered the skin structure closer to its pre-injury state (Fig. S10 and S11). These results indicated desirable properties endowed 8% HA-EPL with the best healing efficiency.

Cytokines are highly correlated with wound healing, which can indirectly reflect the therapeutic effect of dressings and the quality of wound healing [39]. Therefore, some cytokines were selected as indicators of wound healing. Here, tumor necrosis factor-α (TNF-α) was selected to investigate the inflammation of new tissues. Compared with the untreated group, lower expression of TNF-α was observed in the 3 M and HA-EPL groups (Fig. 5d), indicating that both Tegaderm Film and 8% HA-EPL could reduce the inflammatory response. In addition, the reconstruction of blood vessels can provide sufficient oxygen and nutrients for cells to accelerate the wound healing process [40]. Therefore, immunofluorescence staining was performed for platelet endothelial cell adhesion molecule-1 (CD31) and vascular endothelial growth factor (VEGF) to evaluate angiogenesis and vasculogenesis. As expected, the HA-EPL group showed higher expression of VEGF and more pronounced angiogenesis (Fig. 5d and Fig. S12). Subsequently, we performed immunofluorescence staining for collagen I (Col-I) and collagen III (Col-III). Better collagen deposition was found after treatment with the Tegaderm Film and 8% HA-EPL, which illustrated their good ability to promote healing (Fig. S13 and S14). In summary, in terms of relative healing area, inflammatory response, collagen deposition, and vascular reconstruction, 8% HA-EPL exhibited a better ability to promote healing than the other two groups.

### In vivo full-thickness infected wound healing

**Fig. 6:**
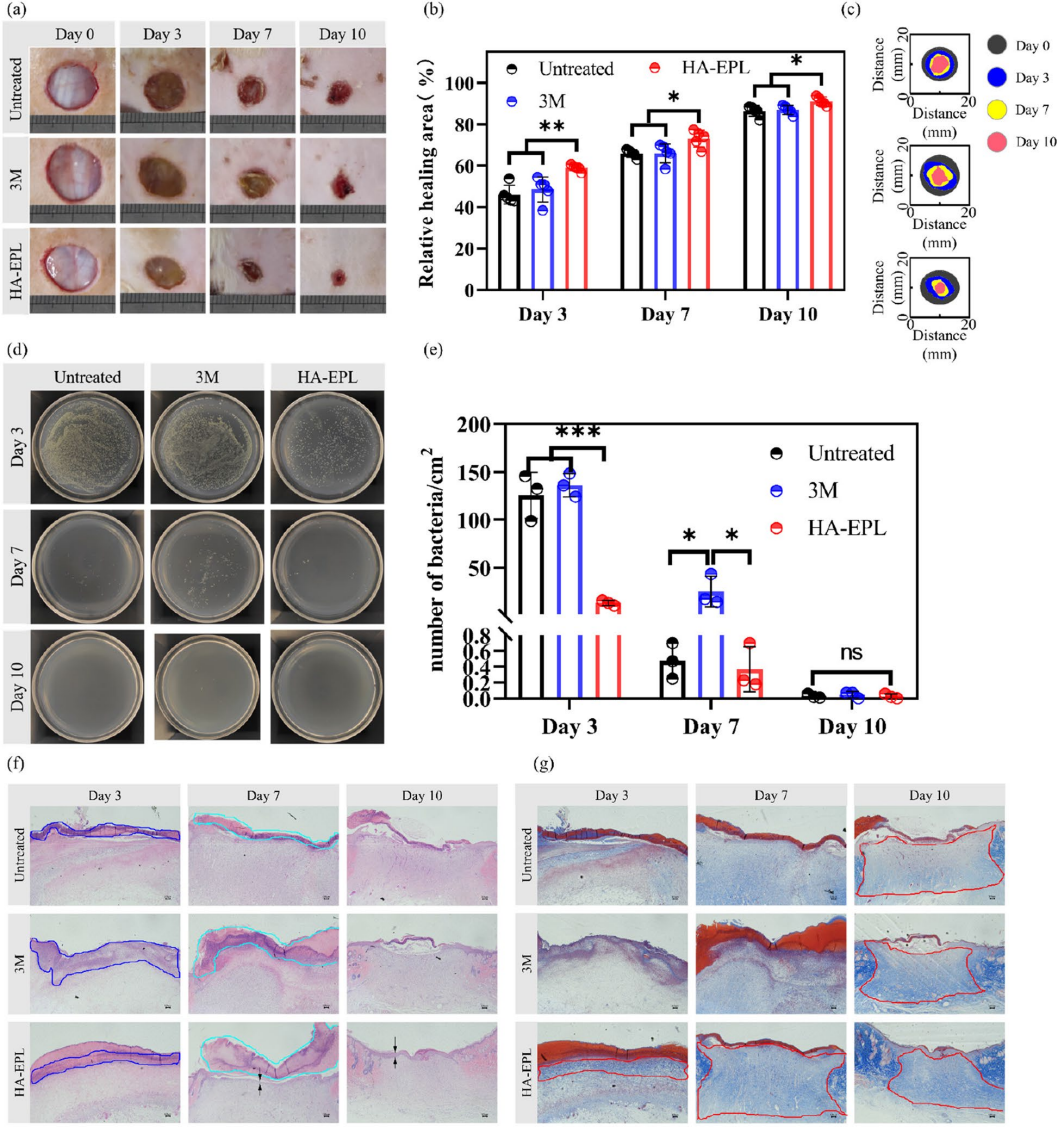
Treatment efficiency of different groups in the full-thickness infected wound. **a** Representative photographs of wounds with different treatments. **b** Quantification of wound contraction rate (*n* = 3). **c** Wound healing track in each group. **d** Representative photographs of the bacterial colony were collected from *S. aureus*-infected wounds. **e** Quantitation of bacterial colonization in the wounds (*n* = 3). **f** H&E staining of wound tissues. Blue frames represent areas of inflammatory infiltration, cyan frames represent residual scar, and black arrows point to the epidermis. **g** Masson staining of wound tissues. The red frames represent the area of collagen deposition at the wound site. ns represents no significant difference, *P < 0.05, **P < 0.01, ***P < 0.001. Scale bar: 100 μm

According to the results of antibacterial tests in vitro and full-thickness wound healing in vivo, 8% HA-EPL showed excellent effects in bacteriostasis and the promotion of wound healing. Besides, *S. aureus* was often found in infected wounds, and many researchers have used *S. aureus* to establish the infection model. Therefore, a full-thickness infected skin defect model was established to evaluate the potential application of 8% HA-EPL. As depicted in Fig. 6a to c, after healing for 3 days, wound contraction in the HA-EPL group was more obvious than that in the other groups (*P* < 0.01). Correspondingly, the number of *S. aureus* colonies in the wound decreased nearly ten-fold compared with the other two groups (Fig. 6d and e; *P* < 0.001), thus demonstrating the remarkable antibacterial ability of 8% HA-EPL. Similarly, on the seventh and tenth days, the therapeutic effect of the HA-EPL group was also better than that of the untreated group and the 3 M group (*P* < 0.05). Furthermore, the 3 M group still showed pathogen contamination (*P* < 0.05), whereas the *S. aureus* was almost cleared in the HA-EPL group. Overall, the antibacterial ability of 8% HA-EPL resulted in a significant difference in wound healing rates compared with other groups.

Similarly, H&E and Masson staining were performed to evaluate the effect of 8% HA-EPL. As displayed in Fig. 6f, on the third day, enhanced inflammatory infiltration was observed in both the untreated and 3 M groups. Comparatively, the inflammatory infiltration was significantly suppressed, and more fibroblasts were gathered around the impaired region. The colonization of bacteria in the wound prolongs the inflammatory period and delays wound healing. The excellent antibacterial activity from 8% HA-EPL was found to eliminate bacteria and downregulate inflammation, which may enter the proliferation period early. This result was also confirmed by Gram staining. On the third day, obvious bacterial colonization was found in the wound bed and the skin appendages (sweat glands, etc.) of normal tissue in both the untreated and 3 M groups, while in the HA-EPL group, the bacterial colonization was significantly reduced and no infection of the appendages was observed (Fig. S15). On the seventh day, inflammatory infiltration remained in the untreated and 3 M groups. However, the inflammatory infiltration disappeared, and the epidermis was visible in the HA-EPL group (Fig. 6f). Additionally, residual scabs were still found in both the untreated and 3 M groups, indicating the delayed healing process of infected wounds. Meanwhile, compared with the untreated and 3 M groups, there was obvious collagen deposition (Fig. 6g), and no obvious signs of infection were found in the HA-EPL group (Fig. S15). After 10 days of treatment, the wound in the HA-EPL group had completely closed, and the epidermis was quite clear compared with the other two groups. Correspondingly, it was found that both the Tegaderm Film group and 8% HA-EPL group exhibited higher collagen deposition than that of the untreated group. In summary, 8% HA-EPL could shorten the healing time of *S. aureus*-infected wounds through efficient sterilization, downregulation of inflammation, and promoting collagen deposition.

**Fig. 7:**
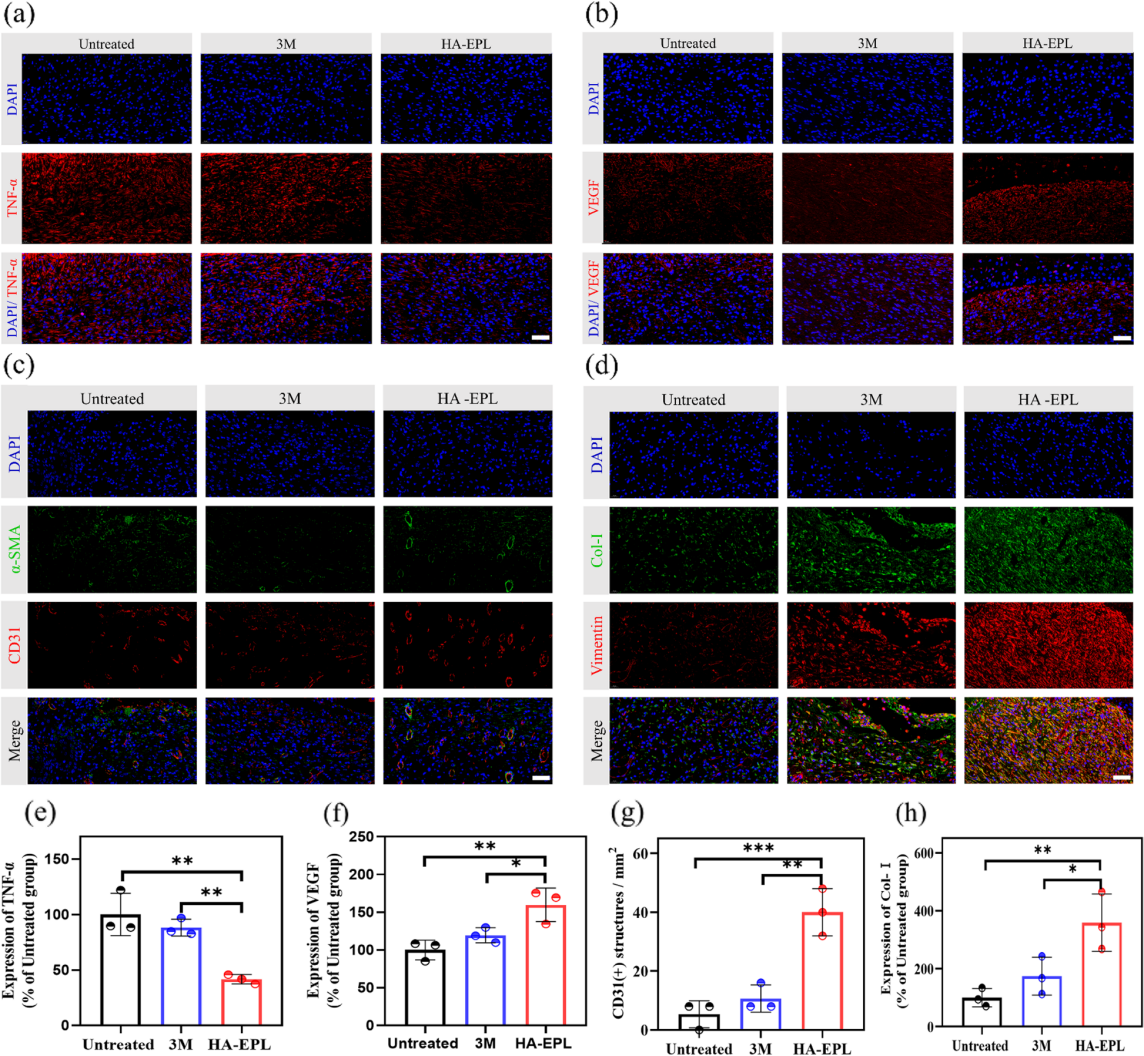
Immunofluorescence staining of different groups in the full-thickness infected wound. **a** Immunofluorescence staining of TNF-α (red) and nuclei (blue) on day 10 in the full-thickness infected wound. **b** Immunofluorescence staining of VEGF (red) and nuclei (blue) on day 10 in the full-thickness infected wound. **c** Double immunofluorescence staining of CD31 (red) and α-smooth muscle actin (α-SMA, green). CD31+ structures (red) were surrounded by α-SMA positive cells (green), implying vascular ducts. **d** Double immunofluorescence staining of collagen I (Col-I, green) and fibroblast marker vimentin (red). **e** Quantification of relative TNF-α expression. **f** Quantification of relative VEGF expression. **g** Quantification of CD31 labeled structures. **h** Quantification of relative collagen expression. *n* = 3, ns represents no significant difference, *P < 0.05, **P < 0.01, ***P < 0.001. Scale bar: 50 μm

The efficacy of 8% HA-EPL in preventing infection was firstly investigated via immunofluorescence staining for TNF-α in the wound bed (Fig. 7a and e). After 10 days of treatment, TNF-α was still detected in the untreated and 3 M groups, indicating a high level of inflammation at the wound site. In contrast, only a small amount of TNF-α was expressed in the HA-EPL group, suggesting that the inflammation and infection of the wound had been improved. This result could be attributed to the presence of EPL in 8% HA-EPL, which has been demonstrated to have broad-spectrum antibacterial properties. Furthermore, revascularization plays an important role in the wound healing process. As shown in Fig. 7b and f, a higher expression of VEGF was observed in the HA-EPL group. Similarly, double immunofluorescence staining of CD31 and α-smooth muscle actin (α-SMA) showed that the density of blood vessels in wound tissue significantly increased in the HA-EPL group (Fig. 7c and g), which illustrated the capability of 8% HA-EPL to recruit host cells for angiogenesis [41]. In wound healing, fibroblasts secrete collagen to enhance the mechanical strength of the new skin. As depicted in Fig. 7d and h, fibroblasts in the HA-EPL group were abundantly recruited at the wound bed, and Masson staining additionally indicated that the collagen deposition was significantly higher than that in the other groups (Fig. 6g). In addition to preventing infection and reestablishing blood vessels, 8% HA-EPL also showed extraordinary advantages for epidermal regeneration and collagen deposition. After 10 days of treatment, the wound in the HA-EPL group had formed an intact epidermis, almost surrounded by a layer of complete keratinocytes. However, in the untreated and 3 M groups, only a few keratinocytes migrated to the wound bed (Fig. S16). Furthermore, the expression of VEGF and inflammation-related genes were tested by q-PCR to demonstrate the advantage of 8% HA-EPL. As shown in Fig. S17, the VEGF expression was significantly up-regulated in the HA-EPL group, and all inflammation factor expression including TNF-α and interleukin-1beta (IL-1β) was down-regulated after 8% HA-EPL treatment. These results suggested that 8% HA-EPL accelerated wound healing through the regulation of inflammation, collagen deposition, pro-vascularization and promoting the regeneration of the new epidermis.

## Discussion

We prepared a new kind of wound dressing with HA and EPL through electrostatic self-assembly. Typically, HA is an acidic polysaccharide and is negatively charged because of the ionized carboxyl groups in the aqueous solution, while EPL is positively charged [42–44]. Benefiting from the strong electrostatic interactions, HA and EPL aggregated rapidly to form HA-EPL coacervates when they were mixed. Infrared spectra confirmed that electrostatic interactions and hydrogen bonds synergistically facilitated the formation of HA-EPL coacervates. These physical interactions made HA-EPL coacervates exhibited a three-dimensional network structure, which was helpful for the exchange of water and air at the wound site. Besides, electrostatic interactions and hydrogen bonds coexist as reversible interactions in the dynamic network of HA-EPL coacervates. Excellent injectability and self-healing ability are endowed to HA-EPL coacervates by destroying and recovering electrostatic interactions and hydrogen bonds. Injectability and self-healing properties allow HA-EPL coacervates to be used conveniently and cover wounds perfectly.

Good adhesion helps the dressing to be anchored to the wound, but it will lead to the inconvenient replacement of the dressing. The emergence of on-demand removal dressing provides a feasible solution to this problem. Given the ability of the salt solution to weaken the electrostatic interactions between HA and EPL, HA-EPL coacervates were predicted to be sensitive to NaCl solution. Therefore, the prepared dressing could be removed by the NaCl solution. 8% HA-EPL on the rat skin and human skin could be quickly dissolved by NaCl-soaked gauze. Meanwhile, SEM images and rheological tests also indicated that the collapsed structure of HA-EPL coacervates when treated with NaCl solution. However, the faster HA-EPL coacervates dissolved, the higher the concentration of NaCl solution needed. The dissolution of HA-EPL coacervates in 20 min required the concentration of NaCl solution to be at least 0.5 M. It is appropriate to deal with the wound with normal saline, while HA-EPL coacervates can be stable for at least 3 days (Fig. S18) after treatment with normal saline according to our experiments. Although it may be more reasonable to use NaCl as the removal medium than to use acid, alkali or special substances to remove dressings [18, 26–28, 45], it is undoubtedly most acceptable for medical staff and patients to use normal saline.

The inherent antibacterial property of EPL also endows HA-EPL coacervates with excellent antibacterial ability. The inhibition effect of HA-EPL coacervates on *S. aureus* was better than that of *E. coli* because of the differences in the cell wall and cell membrane structure between Gram-negative and Gram-positive bacteria. Besides, removing infected skin tissue has been proven to be an effective method of controlling infection and promoting wound healing, but bleeding during the operation remains an inevitable problem. The hemostatic property of HA-EPL coacervates comprised synergistic effects of multiple mechanisms. Firstly, the hemostatic ability of HA-EPL coacervates benefited from their good adhesion and self-healing properties. HA-EPL coacervates could not only adhere well to the bleeding point but also rapidly fused to cover the wound, which served as a physical barrier for hemostasis. Furthermore, the inherent procoagulant activity of EPL also contributed to the hemostasis [46]. All of these hemostatic mechanisms synergistically lead to the good hemostatic ability of 8% HA-EPL, making it a good candidate for medical dressings.

After proving the effect of HA-EPL coacervates on antibacterial and hemostasis in vitro, a full-thickness infected skin defect model was established to evaluate the potential application of 8% HA-EPL. On the one hand, after treatment with 8% HA-EPL, the inflammatory infiltration of the wound was significantly reduced. Wound infection is one of the triggers of the enhanced inflammatory response. After *S. aureus* skin infections, the production of proinflammatory cytokines and other inflammatory mediators (such as IL-1β and TNF) would promote neutrophil recruitment from the bloodstream to form an abscess to facilitate bacterial clearance [39]. Due to the inherent antibacterial activity of EPL in HA-EPL coacervates, the prepared dressing could downregulate inflammatory infiltration by effectively killing bacteria. On the other hand, higher expressions of VEGF and CD31 were observed in the HA-EPL group, indicating its excellent ability to promote angiogenesis and vasculogenesis. It is attributed to HA playing multiple biological roles, including pro-angiogenic activity [42]. In addition, HA assists the invasion and proliferation of fibroblasts, which is necessary for collagen deposition in the wound, as well as promotes the differentiation of fibroblasts into myofibroblasts that play a key role in wound contraction [47, 48]. Therefore, a higher wound contraction rate and better collagen deposition were observed in the HA-EPL group.

In conclusion, the rapid shape adaptability of HA-EPL coacervates enabled them to fit the wound completely, the morphology of the three-dimensional network pore structure in HA-EPL coacervates provided a suitable microenvironment for the proliferation and migration of cells, the on-demand removal property could prevent the adhesion between dressings and new tissues to reduce the pain when changing the dressing, and the excellent antibacterial and hemostatic properties provide favorable conditions for promoting wound healing, thereby accelerating the skin wound healing through the regulation of inflammation, collagen deposition, pro-vascularization and promoting the regeneration of the new epidermis.

## Conclusion

In this study, we successfully designed an injectable and on-demand removal dressing with antibacterial property to facilitate the promotion of bacterial infection wound healing. This dressing was conveniently prepared via electrostatic interactions between HA and EPL. Owing to the reversible nature of electrostatic interactions, HA-EPL coacervates exhibited excellent injectable and self-healing properties, which could substantially facilitate the application of HA-EPL coacervates. In vitro antibacterial experiments showed that HA-EPL coacervates possessed broad-spectrum antibacterial activity against Gram-negative (*E. coli*) and Gram-positive (*S. aureus*) bacteria. In addition, 8% HA-EPL showed good blood-clotting capacity in vitro and hemostatic capacity in vivo. Notably, the salt solution could remove HA-EPL coacervates effortlessly by disrupting the electrostatic interactions. As a wound dressing, 8% HA-EPL exhibited excellent therapeutic effects by inhibiting bacteria, downregulating inflammation, and collagen deposition. Furthermore, the immunofluorescence staining results for CD31, VEGF, K10, and K14 also suggested that 8% HA-EPL has a promotive effect on wound healing by enhancing keratinocyte migration and vascular regeneration. These results indicate that HA-EPL coacervates constitute a simple and practical combined dressing for clinical application.

## Supplementary Information

**Additional file 1: Movie S1.** HA-EPL coacervates could be continuously extruded from the needle.

**Additional file 2: Movie S2.** The process of on-demand removal on the rat wound.

**Additional file 3: Movie S3.** The process of on-demand removal on the human hand.

**Additional file 4: Movie S4.** 8% HA-EPL immediately adhered to the wound surface and sealed the open blood vessels after liver resection.

**Additional file 5: Fig. S1.** (a) FTIR spectra of 4%, 6% and 8% HA-EPL. (b) FTIR spectra of HA, EPL, and 8% HA-EPL. **Fig. S2.** (a) X-ray diffraction spectra of HA, EPL, 4, 6 and 8% HA-EPL. (b) X-ray diffraction spectra of HA, EPL and 8% HA-EPL. **Fig. S3.** 8% HA-EPL could cover various shapes of wounds after extrusion through the needle. **Fig. S4.** Extrusion of 8% HA-EPL through the needle to write a specific letter “XJTU”. **Fig. S5.** 8% HA-EPL adhered firmly to the surface of porcine skin and even stuck two separate pieces of porcine skin together very well. **Fig. S6.** The state of 4, 6 and 8% HA-EPL in different concentrations of NaCl solution. **Fig. S7.** Effects of HA, EPL, and 8% HA-EPL on the heparinized rat blood. **Fig. S8.** The cytotoxicity of 4% HA-EPL, 6% HA-EPL and 8% HA-EPL (*n* = 5). **Fig. S9.** Organ toxicity evaluation of 8% HA-EPL. Scale bar:500 μm. **Fig. S10.** H&E staining of wound tissues. **Fig. S11.** Masson’s trichrome staining of wound tissues. **Fig. S12.** Immunofluorescence staining of CD31 (red) and nuclei (blue) at day 15 in the full-thickness wound. Scale bar: 50 μm. **Fig. S13.** Immunofluorescence staining of Col-I (red) and nuclei (blue) at day 15 in the full-thickness wound. Scale bar: 50 μm. **Fig. S14.** Immunofluorescence staining of Col-III (red) and nuclei (blue) at day 15 in the full-thickness wound. Scale bar: 50 μm. **Fig. S15.** Gram staining of wound tissues. Red frame: bacterial colonization was found in the wound bed. Yellow arrow: bacterial colonization was found in the skin appendages Scale bar: 100 μm. **Fig. S16.** Double immunofluorescence staining of cytokeratin 10 (K10, red) and cytokeratin 14 (K14, green). Scale bar: 50 μm. **Fig. S17.** The gene expression around the full-thickness infected skin wound area was extracted and quantitively evaluated by q-PCR test (*n* = 3). (a) Relative expression of VEGF. (b) Relative expression of TNF-α. (c) Relative expression of IL-1β. *P < 0.05, **P < 0.01, ***P < 0.001. **Fig. S18.** Degradation profile of HA-EPL coacervates in normal saline solution at 37 °C (*n* = 4).

## Data Availability

The datasets used and/or analysed during the current study are available from the corresponding author on reasonable request.

## Abbreviations

HA: Hyaluronic acid
EPL: ε-polylysine
HA-EPL: Hyaluronic acid-ε-polylysine coacervate
FT-IR: Fourier transform infrared spectrometer
XRD: X-ray diffraction
SEM: Scanning electron microscopy
TNF-α: tumor necrosis factor-α
CD31: Platelet endothelial cell adhesion molecule-1
VEGF: Vascular endothelial growth factor
Col-I: Collagen I
Col-III: Collagen III
α-SMA: α-smooth muscle actin
K10: cytokeratin 10
K14: cytokeratin 14
IL-1β: interleukin-1beta
*S. aureus*: *Staphylococcus aureus*
*E. coli*: *E*sch*e*richia *coli*
