# Supplementary material for: A wound-friendly antibacterial hyaluronic acid dressing with on-demand removability for infected wound healing
Source: Biomater Res. 2023 May 1;27:38. doi: 10.1186/s40824-023-00340-7 (PMC10150494; doi:10.1186/s40824-023-00340-7)
Supplement: Supplementary file 5 — Additional file 5: Fig. S1. (a) FTIR spectra of 4%, 6% and 8% HA-EPL. (b) FTIR spectra of HA, EPL, and 8% HA-EPL. Fig. S2. (a) X-ray diffraction spectra of HA, EPL, 4, 6 and 8% HA-EPL. (b) X-ray diffraction spectra of HA, EPL and 8% HA-EPL. Fig. S3. 8% HA-EPL could cover various shapes of wounds after extrusion through the needle. Fig. S4. Extrusion of 8% HA-EPL through the needle to write a specific letter “XJTU”. Fig. S5. 8% HA-EPL adhered firmly to the surface of porcine skin and even stuck two separate pieces of porcine skin together very well. Fig. S6. The state of 4, 6 and 8% HA-EPL in different concentrations of NaCl solution. Fig. S7. Effects of HA, EPL, and 8% HA-EPL on the heparinized rat blood. Fig. S8. The cytotoxicity of 4% HA-EPL, 6% HA-EPL and 8% HA-EPL (n = 5). Fig. S9. Organ toxicity evaluation of 8% HA-EPL. Scale bar:500 μm. Fig. S10. H&E staining of wound tissues. Fig. S11. Masson’s trichrome staining of wound tissues. Fig. S12. Immunofluorescence staining of CD31 (red) and nuclei (blue) at day 15 in the full-thickness wound. Scale bar: 50 μm. Fig. S13. Immunofluorescence staining of Col-I (red) and nuclei (blue) at day 15 in the full-thickness wound. Scale bar: 50 μm. Fig. S14. Immunofluorescence staining of Col-III (red) and nuclei (blue) at day 15 in the full-thickness wound. Scale bar: 50 μm. Fig. S15. Gram staining of wound tissues. Red frame: bacterial colonization was found in the wound bed. Yellow arrow: bacterial colonization was found in the skin appendages Scale bar: 100 μm. Fig. S16. Double immunofluorescence staining of cytokeratin 10 (K10, red) and cytokeratin 14 (K14, green). Scale bar: 50 μm. Fig. S17. The gene expression around the full-thickness infected skin wound area was extracted and quantitively evaluated by q-PCR test (n = 3). (a) Relative expression of VEGF. (b) Relative expression of TNF-α. (c) Relative expression of IL-1β. *P < 0.05, **P < 0.01, ***P < 0.001. Fig. S18. Degradation profile of HA-EPL coacervates in normal [file 40824_2023_340_MOESM5_ESM.docx]

Supporting Information

A Wound-Friendly Antibacterial Hyaluronic Acid Dressing with On-Demand Removability for Infected Wound Healing

*Datao Hu^1,†^, Jinpeng Wen^1,†^, Xinxin Zhao^1^, Kailai Liu^1^, Yuchen Zhang^1^, Yizhuo Bu^1^ and Ke Wang^1, 2,*^*

^1^ School of Pharmacy, Health Science Center, Xi’an Jiaotong University, Xi’an 710061, China

^2^ State Key Laboratory of Natural and Biomimetic Drugs, Peking University

*Correspondence to [perpetual1003@mail.xjtu.edu.cn](mailto:perpetual1003@mail.xjtu.edu.cn)

^†^These authors contributed equally to this work.

**Methods**

**Characterization of HA-EPL coacervates**

The functional groups of HA, EPL and HA-EPL coacervates were determined by a Fourier transform infrared (FT-IR) spectrometer (TENSOR27, Bruker, Germany) for spectroscopy measurement in the range of 4000–400 cm^−1^ with a resolution of 2 cm^-1^. Besides, the crystallinity and amorphous structure of samples were further analyzed by X-ray diffraction (XRD6100, ZHONGHE Instruments) with Cu-Ka radiation. The XRD spectra were recorded in the range of 5°-80° (2θ) with a scan rate of 4°/min. The morphologies of HA-EPL coacervates were observed by a scanning electron microscope (S4800, Hitachi, Japan).

**Rheological tests**

The rheological tests of HA-EPL coacervates were performed using an Anton-Paar rheometer (MCR302, Austria) with 20 mm flat plates and 1.0 mm gap at 37 ℃. Firstly, stress sweep tests were conducted at a constant frequency of 1 Hz and a strain range of 0.1% ~ 100% to evaluate the storage modulus (*G′*) and loss modulus (*G″*) of HA-EPL coacervates. Following, time sweep tests were performed with a constant strain of 1% and a constant frequency of 1 Hz. Subsequently, frequency sweep tests were carried out at a constant strain of 1% with shear frequency ranging from 0.1 to 35 Hz. Finally, shear viscosity tests of HA-EPL coacervates were performed by adjusting the shear rate from 0.1 to 1000 s^-1^. Besides, the step-strain-sweep was performed under the alternate conversion between low shear strains (γ = 0.5%) and high shear strains (γ = 500%) with 100 s for each cycle.

**Self-healing and adhesive properties**

Macroscopical self-healing changes of coacervates were observed. Briefly, two groups of heart-shaped 8% HA-EPL were cut into two pieces, and one group was dyed with methyl orange. After that, the two groups of 8% HA-EPL were cut in the middle, and half of each was taken and gently glued together. No extra force was applied to make them fuse, and the self-healing situation was observed. In addition, the tissue adhesion property of 8% HA-EPL was assessed using porcine skin and the main organs of mice. The pre-stained 8% HA-EPL was attached to the surface of a 2×2 cm fresh porcine skin. Afterward, the porcine skin was cut into two parts, and 8% HA-EPL was smeared on the incision. Moreover, they were smeared on the main organs of mice (heart, liver, spleen, lung, and kidney) to evaluate their adhesion property.

**The dissolution behavior of HA-EPL coacervates**

4%, 6% and 8% HA-EPL were immersed in 0.5 M, 1 M and 2 M NaCl solutions and their dissolution behaviors were observed at predetermined times (0 h, 0.5 h, 2 h). The dissolution behaviors of 4%, 6% and 8% HA-EPL were also analyzed by the rheological tests. Briefly, 4%, 6% and 8% HA-EPL were exposed to 0.5 M, 1 M and 2 M NaCl solutions (2 mL) for 30 min. Then, time sweep tests were performed with a constant strain of 1% and a constant frequency of 1 Hz. Furthermore, the morphologies of 4%, 6% and 8% HA-EPL were observed before and after being treated with 1 M NaCl solution for 20 min.

***In vitro* degradation behavior of HA-EPL coacervates** For the in vitro degradation test, HA-EPL coacervates with the same weight (1.0 g) were immersed in 20 mL of normal saline at constant temperature (37 °C) with shaking at 120 rpm, respectively. At the predetermined time point, HA-EPL coacervates were taken out and weighed after removing excess water using dry filter paper. The weight remaining ratio of samples was defined by the following equation:

$$Weight remaining ration \left( \% \right)= \frac{w_{t}}{w_{0}} \times100\%$$

*W*_t_ and *W*_0_ are the weight of remaining samples after degradation at different time points and the initial weight of the samples, respectively.

***In vitro* antibacterial activity evaluation**

The surface antibacterial activities of HA-EPL coacervates for *Escherichia coli* (ATCC 8739) and *Staphylococcus aureus* (ATCC 12228) were evaluated[1]. The bacteria were firstly seeded in fresh LB medium and cocultured with shaking at 120 rpm at 37 ◦C for 6 ~ 8 h, respectively. After that, the bacteria were counted and diluted to the testing concentration to assess the antibacterial properties. HA-EPL coacervates (500 μL) were added to the corresponding centrifuge tube (2 mL). Then, the bacterial suspension (10 μL, 1×10^4^ CFU mL^-1^) was placed onto the surface of HA-EPL coacervates and incubated at 37 °C for 1 h. After that, sterilized phosphate buffer (PBS, 1 mL) was added to the centrifuge tube to re-suspend any bacterial survivor. Next, the bacterial suspension (in PBS, 20 μL) was incubated overnight at 37 °C on plate count agar (PCA). HA-EPL coacervate without addition was used as the control. After overnight incubation, the colony-forming units (CFU) were counted. Tests were repeated in triplicate for each group and the kill rate was calculated by the following equation:

$$\text{kill rate }\left( \text{\%} \right)\text{ = }\frac{\text{the CFU of control group- the CFU of HA}\text{-}\text{EPL group }}{\text{the CFU of control group}}\times100$$

***In vitro* cytotoxicity of HA-EPL coacervates**

Cell culture conditions: L929 cells were cultured in Dulbecco’s Modified Eagle’s Medium (DMEM, Servicebio, China) with 10% fetal bovine serum (FBS, Servicebio, China) and 1% penicillin/streptomycin (Servicebio, China). All cells were maintained at 37 ℃ with 5% CO_2_.

MTT assay: The cytotoxicity of 4% HA-EPL, 6% HA-EPL and 8% HA-EPL to L929 cells was detected by MTT assay. Briefly, L929 cells were seeded in a 96-well plate with a density of 1 × 10^4^ cells per well with 100 μL DMEM. After 24 h incubation, the culture medium was aspirated from the cells and 100 μL of fresh DMEM medium containing different concentrations of samples were added. After incubation for 24 h, carefully remove the culture medium from the plates. 50 μL of MTT solutions were added to each test well and the plates were further incubated for 2 h in the incubator at 37 °C. Then MTT solutions were discarded and 100 μL of isopropanol were added to each well. Sway the plate and subsequently transfer it to a microplate reader to read the absorbance at 570 nm (Bio-Rad, Berkeley, CA).

**Animals**

Sprague-Dawley (SD) (male, 200 ± 10 g) rats and Kunming mice (male, 35 ± 2 g) were purchased from the Laboratory Animal Center of Xi’an Jiaotong University. All animal experiments were approved by the institutional review board of Xi’an Jiaotong University. All experiments followed the Guidelines for the Care and Use of Laboratory Animals of the Ethical Committee of Xi’an Jiaotong University, Xi’an, China (permit No. XJTU 2019-003).

***In vitro* and *in vivo* hemostasis ability**

*In vitro* blood coagulation test: Heparinized rat blood was prepared to investigate the hemostasis ability of HA, EPL, and 8% HA-EPL. Heparinized rat blood (2 mL) was mixed with 4% HA solution, 8% EPL solution, and 8%HA-EPL in the corresponding tube at room temperature for 15 minutes. Then, the coagulation efficacy of samples was evaluated *via* a tube reversion test[2].

*In vivo* hemostasis test: To further evaluate the hemostasis ability of HA-EPL coacervates, the mouse-tail amputation model and acute liver hemostasis model were performed, respectively[3]^.^ For the mouse-tail amputation model, the mice (Kunming mice, male, 35 ± 2 g) were anesthetized by isoflurane and thirty percent length of the tail was cut with surgical scissors. Afterward, the bleeding site was covered with 8% HA-EPL, while the bleeding site without treatment was taken as a control. And the lost blood within 15 seconds was recorded with filter paper. All experiments were tested in triplicate in each group.

For the acute liver hemostasis test, Sprague-Dawley (SD) rats (8 weeks, male, 200 ± 10 g) were anesthetized by isoflurane and fixed on a surgical corkboard. An acute wound of the liver was formed with surgical scissors after exposure of the liver through an abdominal incision, and 8% HA-EPL (500 µL) was injected onto the bleeding site after injury. Meanwhile, a pre-weight filter paper with a hydrophobic membrane was placed beneath the liver. The bleeding site without treatment was used as the control group. The blood loss within 10 seconds of each rat was recorded by video and quantified by the weighting method.

***In vivo* healing of full-thickness wounds**

The therapeutic effect of 8% HA-EPL was evaluated using a mouse full-thickness skin defect model. In brief, the Kunming mice were anesthetized and the dorsal hair was shaved. A full-thickness round wound (10 mm) was inflicted on the shaved back using a punch biopsy. The wound was treated with 8% HA-EPL, 3M (Tegaderm Film, USA), or left undressed, namely HA-EPL, 3M, and untreated. Afterward, the bandage was applied above the 8% HA-EPL and 3M to keep dressings stable. For wound monitoring, wound areas were photographed and measured on 5, 10 and 15 days. The wound areas in each group were calculated using Image J software.

***In vivo* healing of full-thickness infected wounds**

The procedure of a full-thickness infection model is similar to that of the full-thickness defect model. Briefly, three 13 mm diameter full-thickness round defects were created in the center of the shaved back of SD rats (male, 250~280 g). Then, 80 μL of *S. aureus* (10^8^ CFU mL^-1^) was added to the wound to form the infection, and the dressing was covered on the wound after 1 h. Three wounds of each rat were treated with control (untreated group), 3M (3M group), and 8% HA-EPL (HA-EPL group). Wound areas were photographed on 3, 7 and 10 days and measured by Image J software.

In order to evaluate the bacterial infection condition in each group, bacterial samples were collected from wounds with sterilized cotton swabs in the above schedule[4]. And the swabs were put into normal saline solution (1 mL) and diluted 100 times. The diluted bacterial suspension was cultured overnight on PCA at 37 ℃.

**Histological evaluation**

To further investigate the mechanism of 8% HA-EPL on wound healing, the wound tissues were collected at predetermined times and fixed with 4% paraformaldehyde, embedded in paraffin, cross sectioned to 5 μm thickness slices. The slices were subjected to Hematoxylin and Eosin (H&E) staining, Masson’s trichrome staining, and Gram staining. Besides, mice treated with 8% HA-EPL were sacrificed on day 10 and the heart, kidney, spleen, liver and lung were collected for H&E staining.

**Immunofluorescent staining**

The wound tissues were also collected for immunofluorescence staining. The fixed sections were stained with TNF-α (cat. no. GB11188, Servicebio, China), VEGF (cat. no. GB13034, Servicebio, China), CD31 ((cat. no. GB11315-1, Servicebio, China), Col-Ⅰ (cat. no. GB11022-3, Servicebio, China), Col-Ⅲ (cat. no. GB13023-2, Servicebio, China), cytokeratin 10 (K10; cat. no. GB112105, Servicebio, China), cytokeratin 14 (K14; cat. no. GB11803, Servicebio, China), vimentin (cat. no. GB12192. Servicebio, China), and α-smooth muscle actin (α-SMA; cat. no. GB111364, Servicebio, China), respectively. These images were taken by a fluorescence microscope (NIKON ECLIPSE TI-SR, Japan).

**Quantitative real-time PCR**

Quantitative real-time PCR was performed on the wound tissues. The wound tissues of infected rats on day 10 were collected. The RNA was obtained by using the Trizol reagent. Then, cDNA was synthesized by reverse transcription of RNA according to the manufacturer's protocols. The quantitative real-time PCR (q-PCR) was detected with a Real-time fluorescence quantitative PCR instrument (MX3005P, Agilent, USA), and GAPDH was used as the housekeeping gene. The q-PCR primers used for VEGF were 5*'*–TGT GAG CCT TGT TCA GAG CG-3*'* and 5*'*–GGT CTA GTT CCC GAA ACC CTG A-3*'*; TNF-α were 5*'*–CCA CCA CGC TCT TCT GTC TAC TG-3*'* and 5*'*–TGG GCT ACG GGC TTG TCA CT-3*'*; IL-1β were 5*'*–GAA CAA CAA AAA TGC CTC GTG C-3*'* and 5*'*–GAC AAA CCG CTT TTC CAT CTT CT-3*'*.

**Results**

**Movie S1** HA-EPL coacervates could be continuously extruded from the needle.

**Movie S2** The process of on-demand removal on the rat wound.

**Movie S3** The process of on-demand removal on the human hand.

**Movie S4** 8% HA-EPL immediately adhered to the wound surface and sealed the open blood vessels after liver resection.


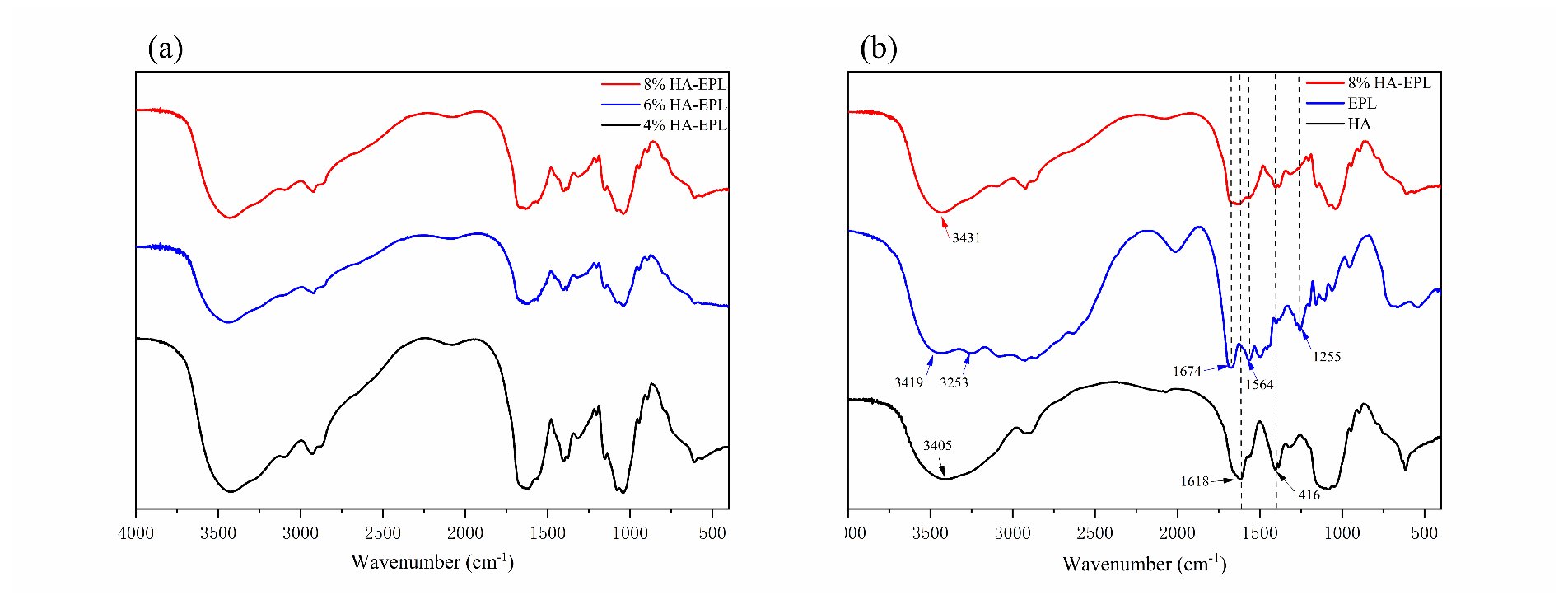


**Figure S1.** (a) FTIR spectra of 4%, 6% and 8% HA-EPL. (b) FTIR spectra of HA, EPL, and 8% HA-EPL.

Electrostatic interactions and hydrogen bonds synergistically facilitated the formation of HA-EPL coacervates by FTIR analysis. The FTIR spectra of HA, EPL and 8% HA-EPL were presented in Figure S1. In the spectra of HA, a broad peak was observed at 3417 cm^-1^ assigned to the stretching vibration of O-H. Also, other notable peaks appeared at 1618 cm^-1^ and 1413 cm^-1^ representing principally C=O stretching vibration in carboxyl group. Synchronously, the characteristic bands of EPL were those of amide I (1674 cm^-1^), II (1564 cm^-1^), and III (1277 cm^-1^), respectively. Other peaks such as 3419 cm^-1^ and 3253 cm^-1^ were assigned to the stretching vibration of N-H.

The spectra of 4%, 6%, and 8% HA-EPL were quite similar to that of HA, represented by the peaks at approximately 3500 cm^-1^ due to the O-H and N-H stretching vibration and at 1700~1200 cm^-1^ due to the amide Ⅰ, Ⅱ and Ⅲ. In the spectrum of 8% HA-EPL, peaks at 1413 cm^−1^ and 1618 cm^−1^ which were related to the stretching vibration of the carboxylic group weakened or disappeared because HA involved in the formation of HA-EPL coacervates. Besides, a decrease in the intensity of bands associated with amide Ⅰ, Ⅱ, and Ⅲ (1674 cm^-1^, 1564 cm^-1^, and 1277 cm^-1^) was also observed. This change might be attributed to electrostatic interactions between the amino groups of EPL and the carboxyl groups of HA during the formation of HA-EPL coacervates. In addition to electrostatic interactions, the O-H stretching band in HA (3417cm^-1^) and the N-H stretching band in EPL (3419 cm^-1^) were shifted to 3431cm^-1^, meaning that hydrogen bonds formed in the formation process of HA-EPL coacervates. Thus, electrostatic interactions and hydrogen bonds together promoted the formation of HA-EPL coacervates.

**
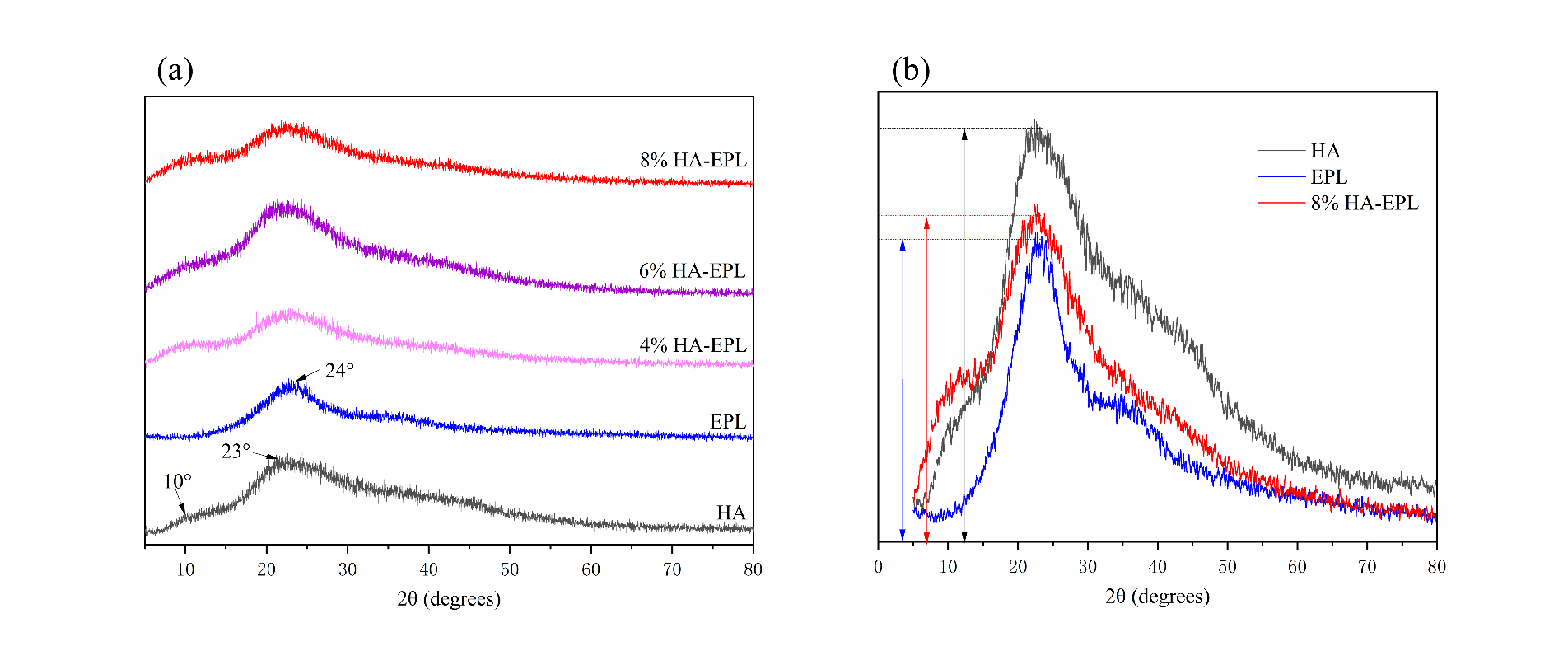
**

**Figure S2.** (a) X-ray diffraction spectra of HA, EPL, 4%, 6% and 8% HA-EPL. (b) X-ray diffraction spectra of HA, EPL and 8% HA-EPL.

According to the X-ray diffraction pattern, we can determine the crystalline or amorphous properties of HA, EPL and HA-EPL coacervates. As shown in **Figure S2a**, a broad peak was observed in the XRD patterns of HA (2θ=23°), EPL (2θ=24°), and HA-EPL coacervates (2θ=24°), corresponding to the amorphous. Besides, for HA-EPL coacervates, the intensity of diffraction peak at 24°(2θ) significantly decreased compared with that of HA as well as slightly increased compared with EPL (Figure S2b). This result showed that the structure of HA and EPL changed during the coacervation process. Previous studies have also reported structural changes caused by physical interactions between protein and polysaccharide. Overall, HA-EPL coacervates were characterized by injectability, self-healing and on-demand removal properties, benefiting from the dynamic interactions between HA and EPL through hydrogen bonds and electrostatic interactions.


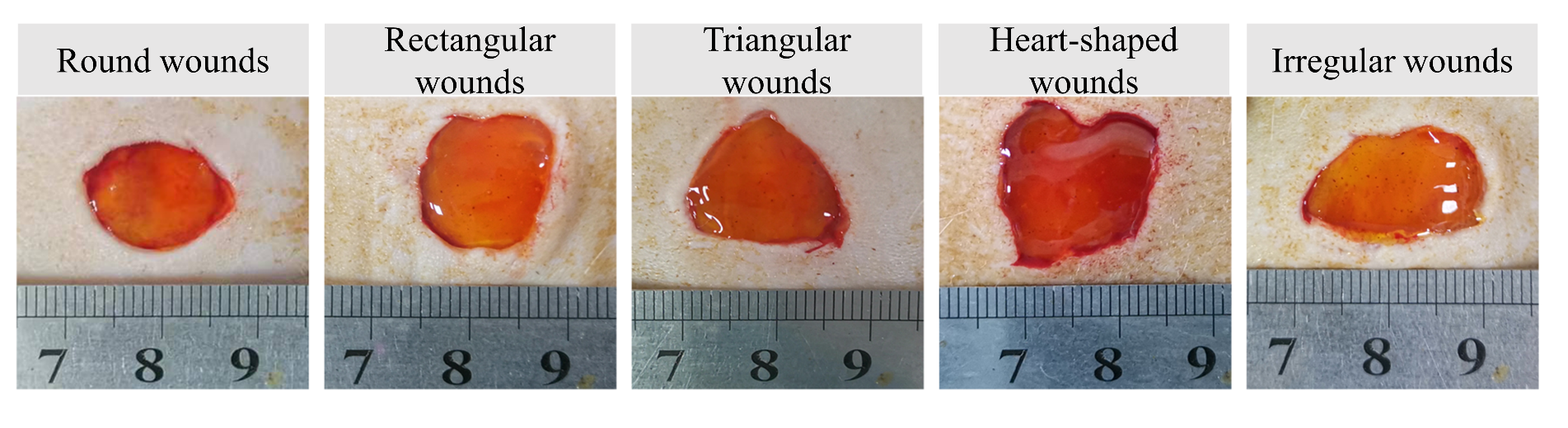


**Figure S3.** 8% HA-EPL could cover various shapes of wounds after extrusion through the needle.


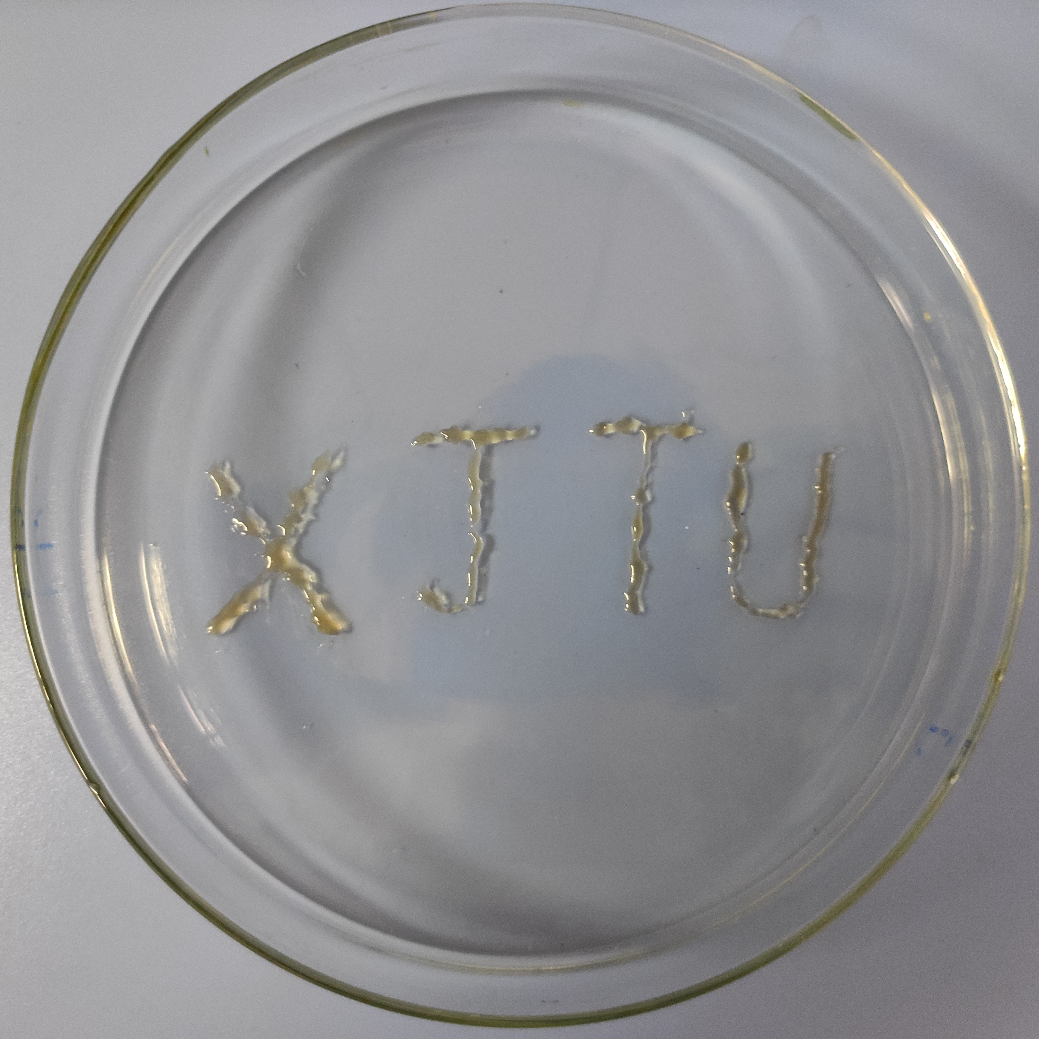


**Figure S4.** Extrusion of 8% HA-EPL through the needle to write a specific letter “XJTU”.


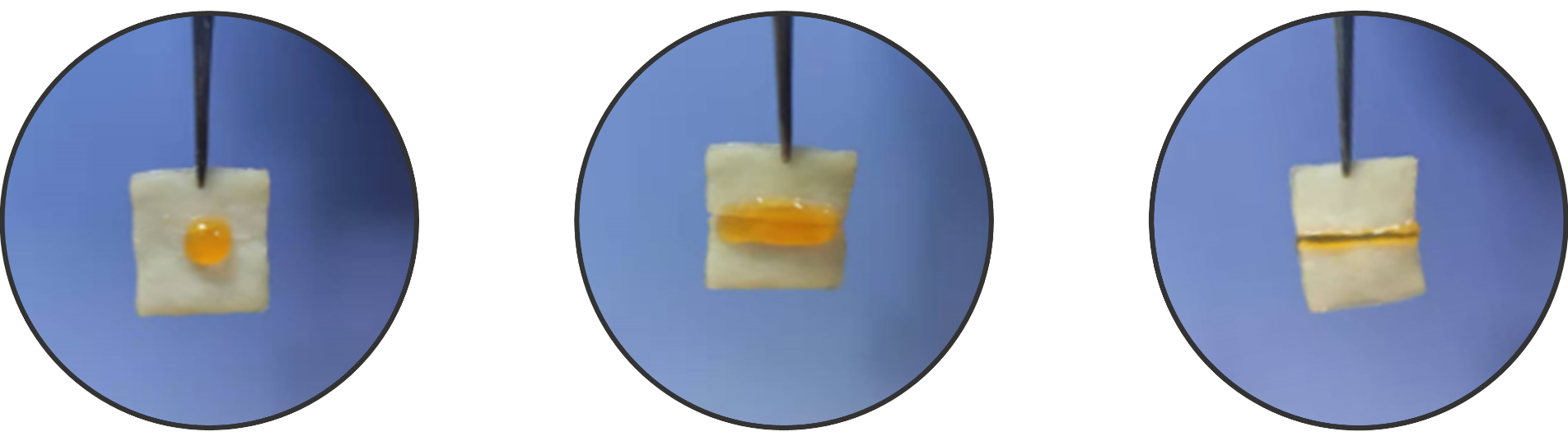


**Figure S5.** 8% HA-EPL adhered firmly to the surface of porcine skin and even stuck two separate pieces of porcine skin together very well.


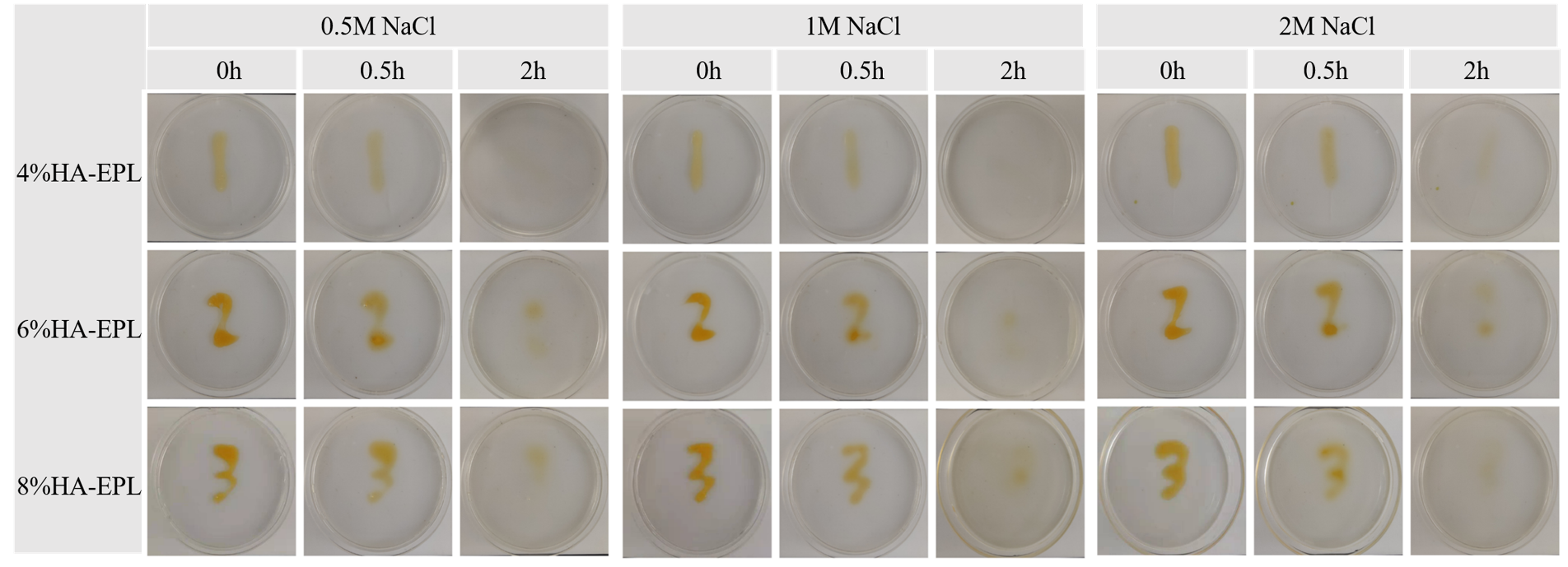


**Figure S6.** The state of 4%, 6% and 8% HA-EPL in different concentrations of NaCl solution.


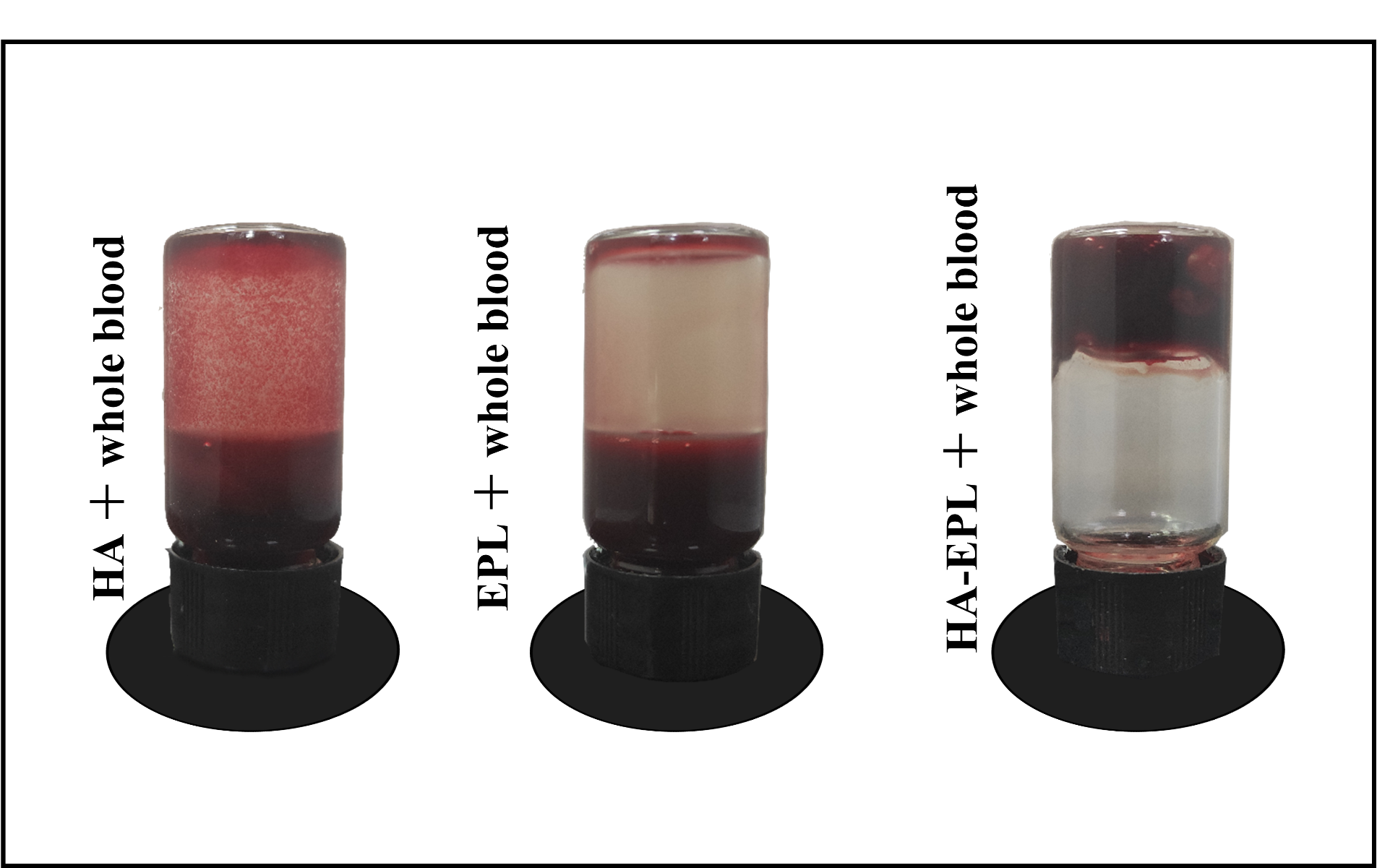


**Figure S7.** Effects of HA, EPL, and 8% HA-EPL on the heparinized rat blood.

**Figure S8.** The cytotoxicity of 4% HA-EPL, 6% HA-EPL and 8% HA-EPL (n = 5).


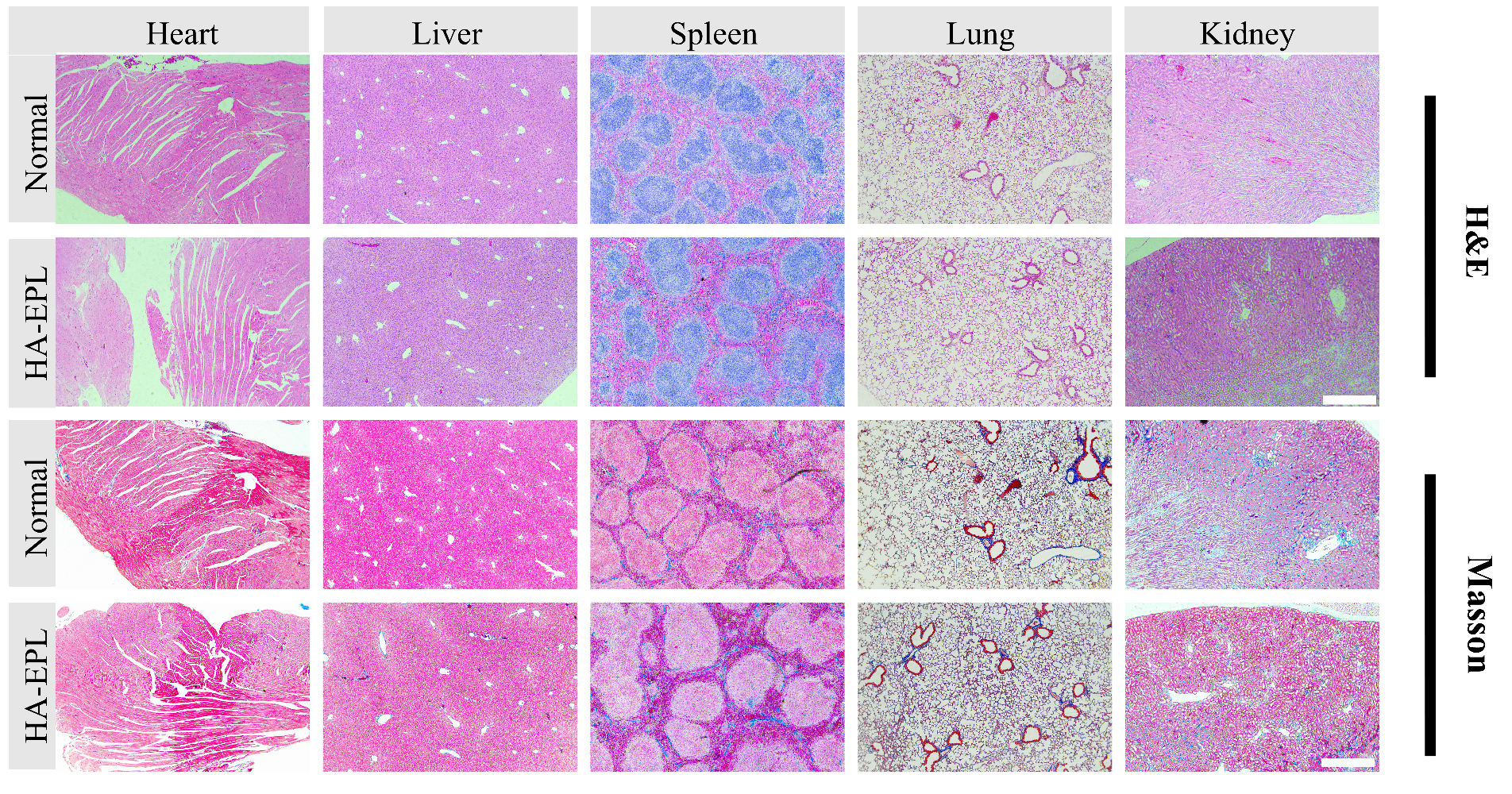


**Figure S9.** Organ toxicity evaluation of 8% HA-EPL. Scale bar:500 μm.


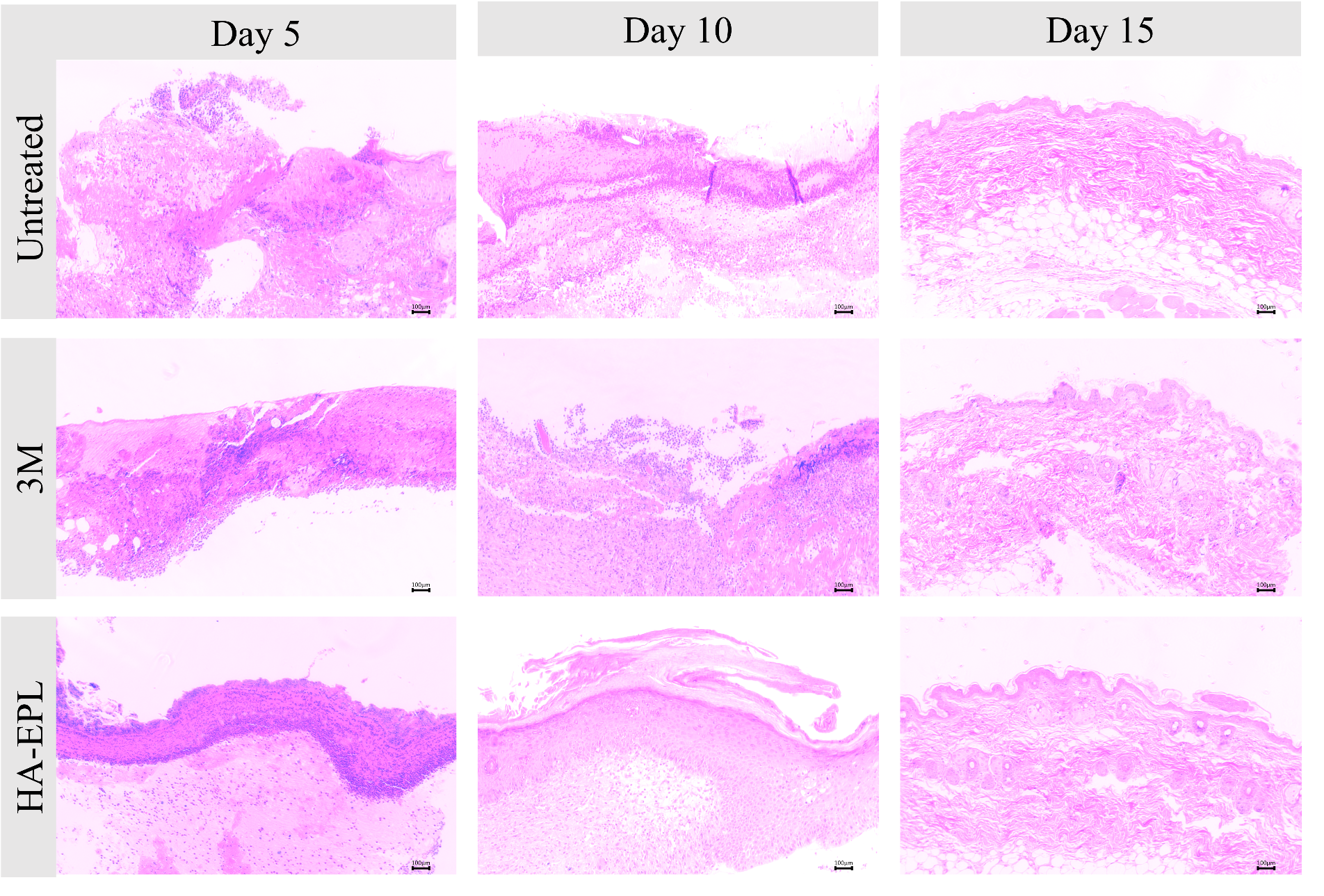


**Figure S10.** H&E staining of wound tissues.


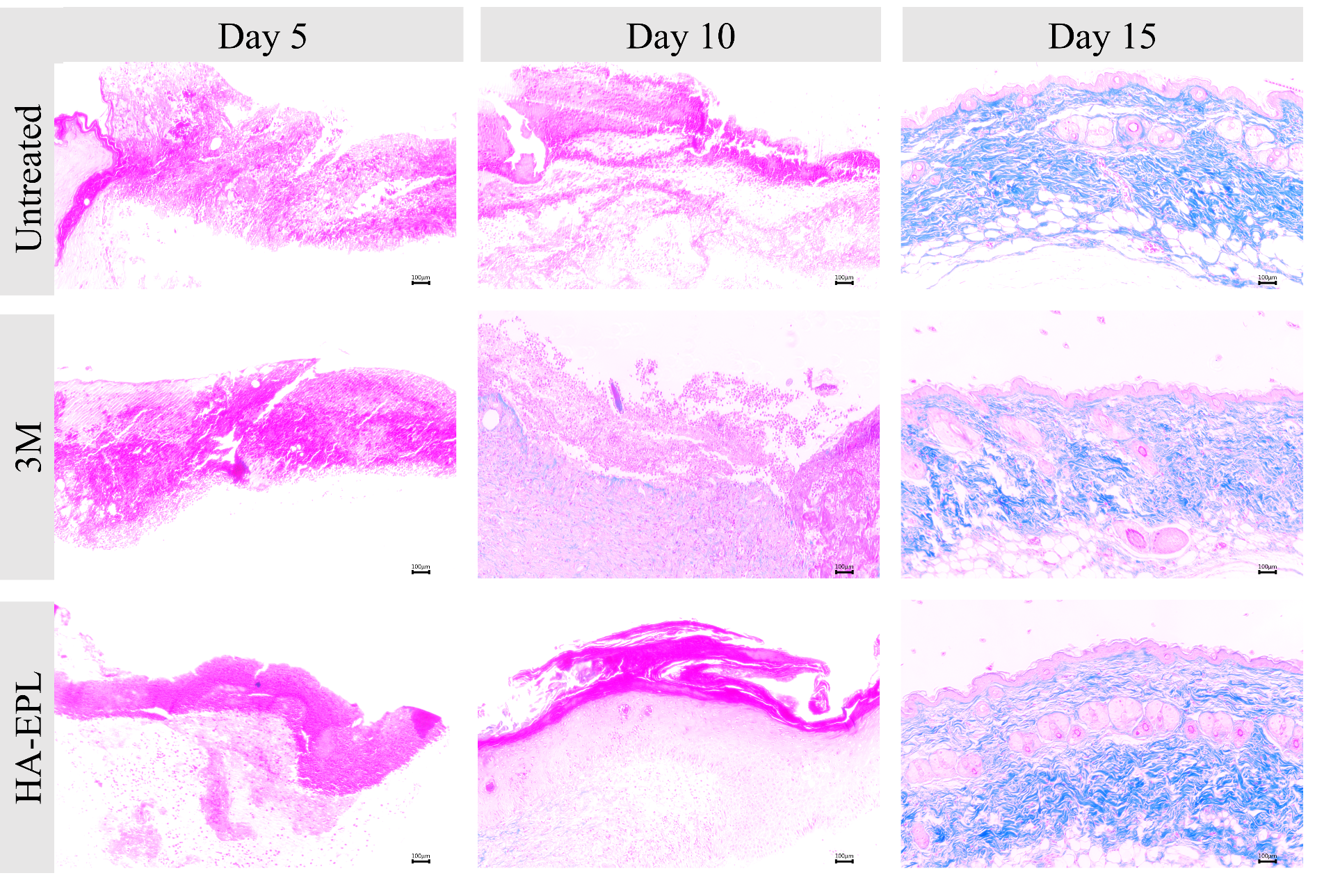


**Figure S11.** Masson’s trichrome staining of wound tissues.

The therapeutic effect of 8% HA-EPL was further evaluated by H&E and Masson staining. As shown in Figure S10, On the 5^th^ day, inflammatory infiltration and incomplete skin structure appeared in all three groups, while the wound bed showed better tissue regeneration ability after 8% HA-EPL treatment. On the 10^th^ day, a large amount of inflammatory infiltration was still present in the untreated group and 3M group. On the contrary, inflammatory infiltration was hardly observed in the HA-EPL group and the structure of the epidermis was clearly visible, indicating that 8% HA-EPL could downregulate inflammation infiltration and promote healing. On the 15^th^ day, some skin appendages (sebaceous glands) appeared in the HA-EPL group compared with the untreated group and 3M group, demonstrating that 8% HA-EPL could not only accelerate wound healing but also reconstruct the structure of damaged skin. Correspondingly, the Masson staining results also showed the wounds treated with 8% HA-EPL presented a highly ordered fibrillar collagen structure and more skin appendages (Figure S11). The results of H&E staining and Masson staining suggested that 8% HA-EPL effectively downregulated inflammation and recovered the skin structure closer to its pre-injury state.


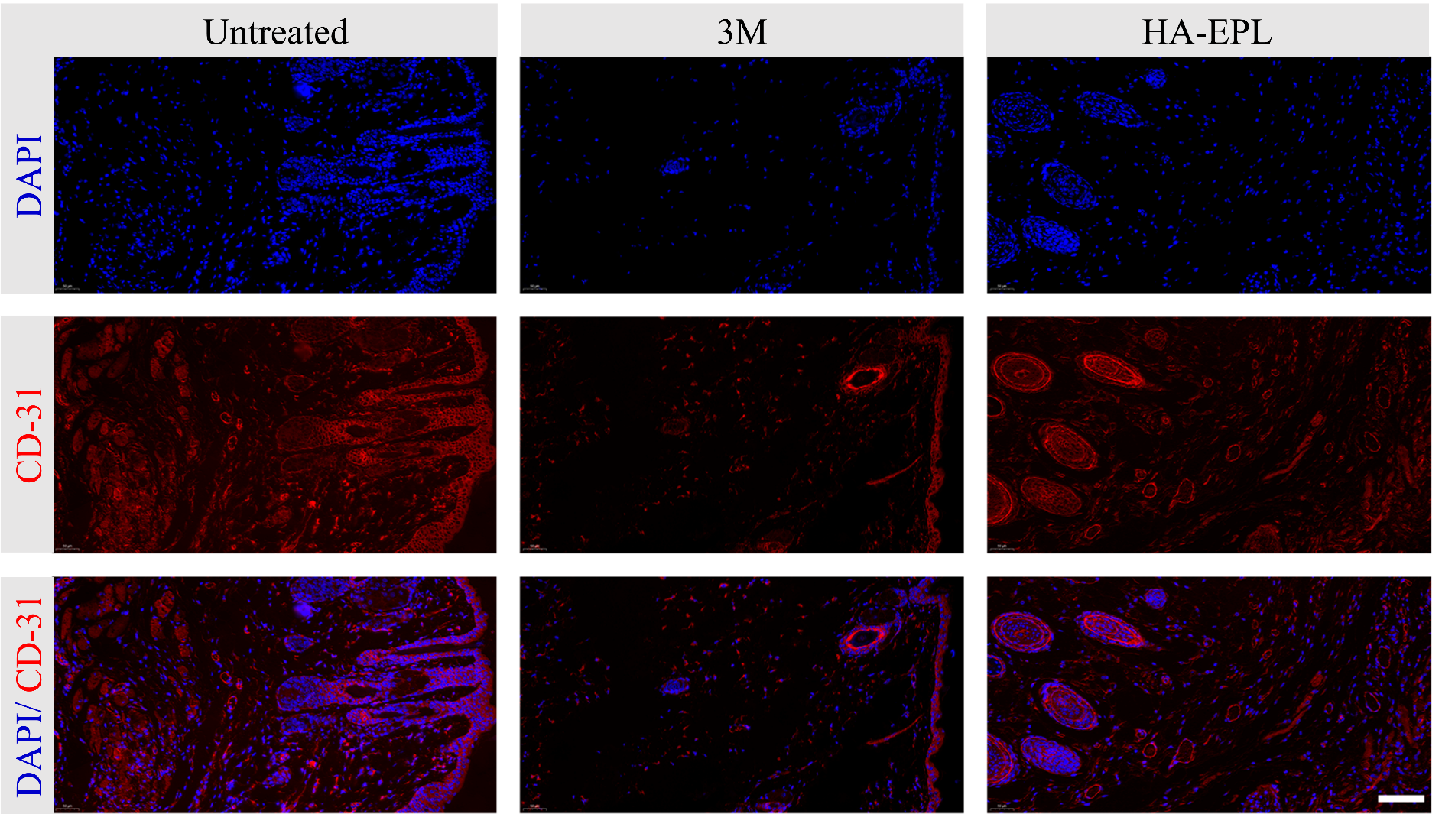


**Figure S12.** Immunofluorescence staining of CD31 (red) and nuclei (blue) at day 15 in the full-thickness wound. Scale bar: 50 μm.


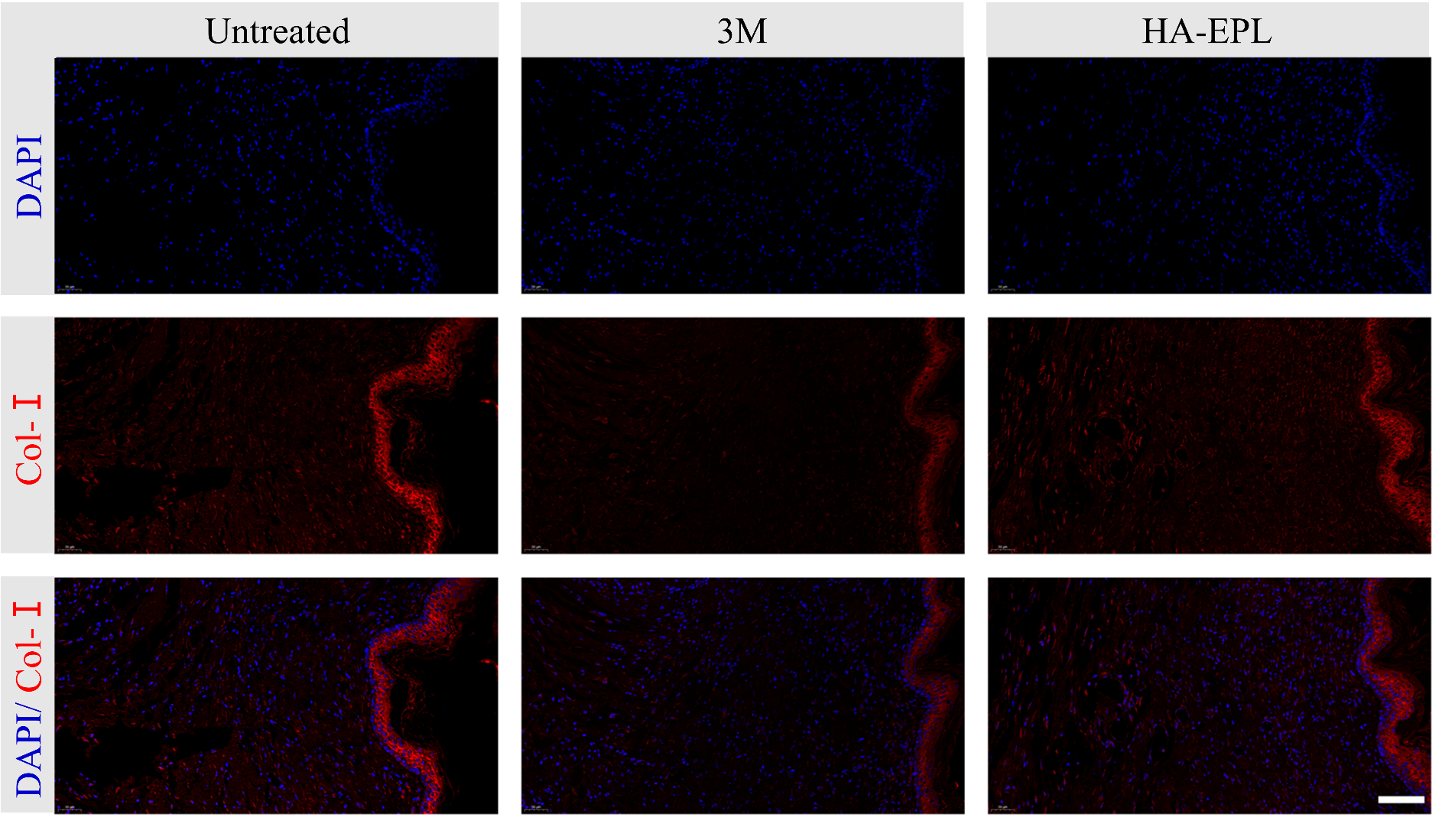


**Figure S13.** Immunofluorescence staining of Col-Ⅰ (red) and nuclei (blue) at day 15 in the full-thickness wound. Scale bar: 50 μm.


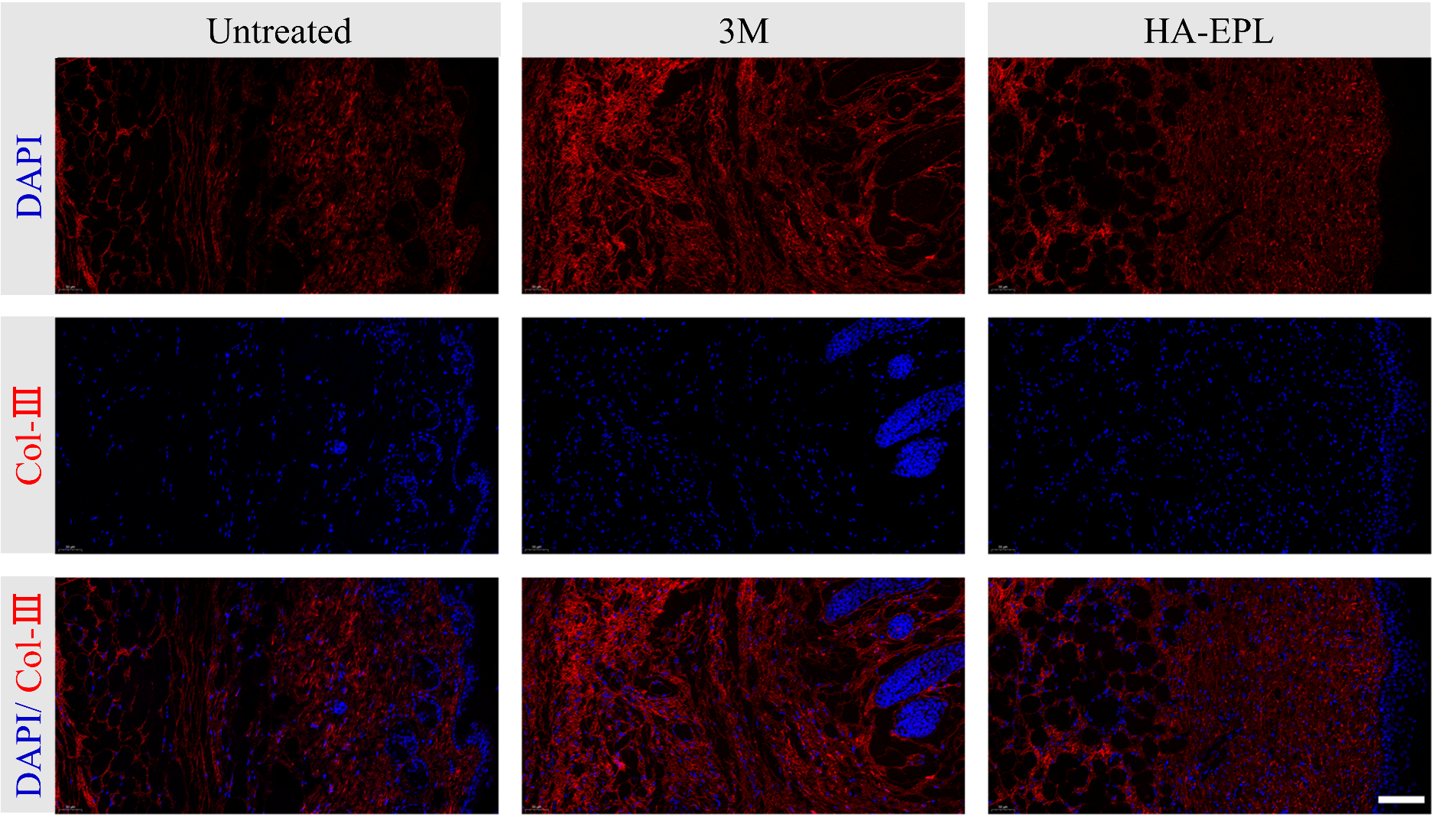


**Figure S14.** Immunofluorescence staining of Col-Ⅲ (red) and nuclei (blue) at day 15 in the full-thickness wound. Scale bar: 50 μm.


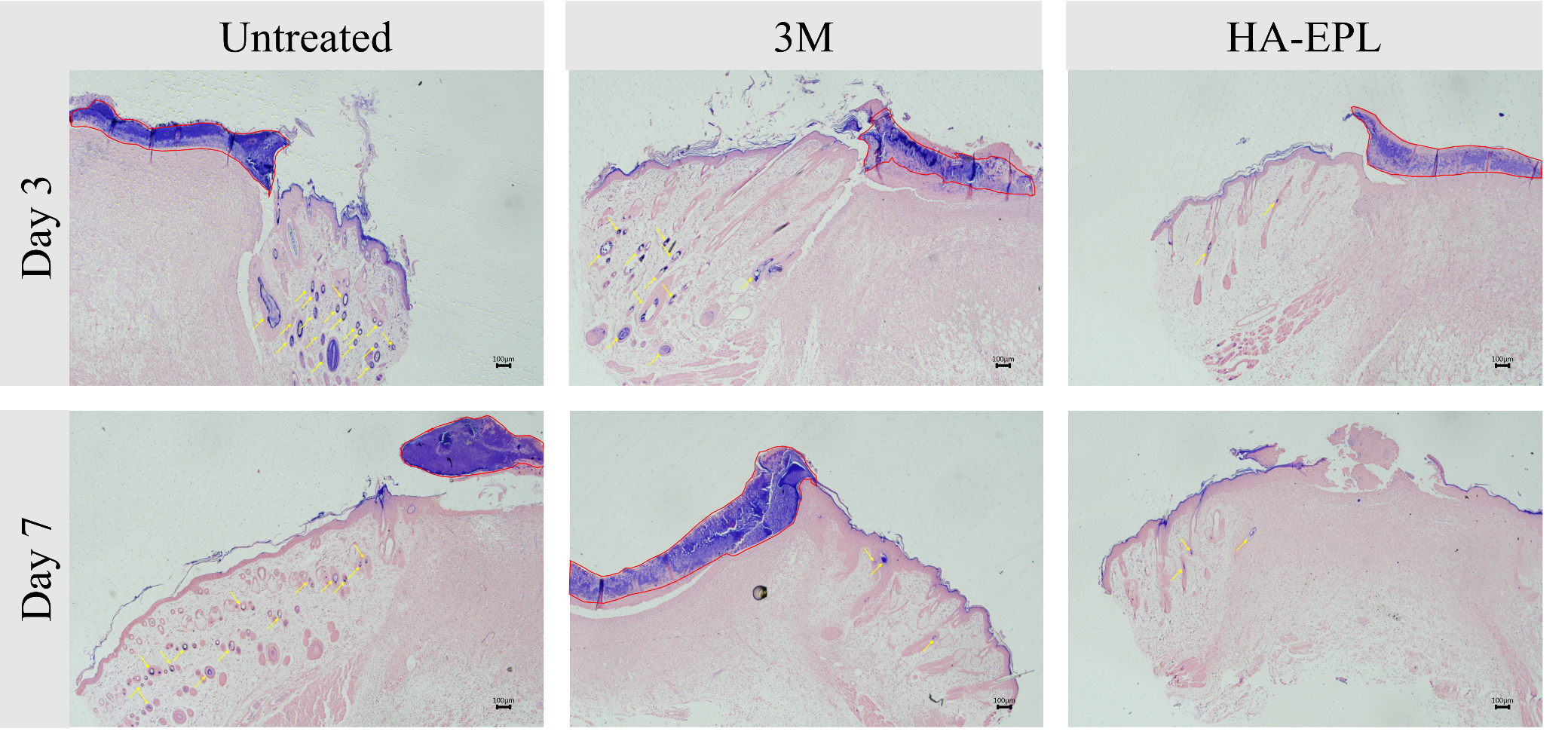


**Figure S15.** Gram staining of wound tissues. Red frame: bacterial colonization was found in the wound bed. Yellow arrow: bacterial colonization was found in the skin appendages Scale bar: 100 μm.


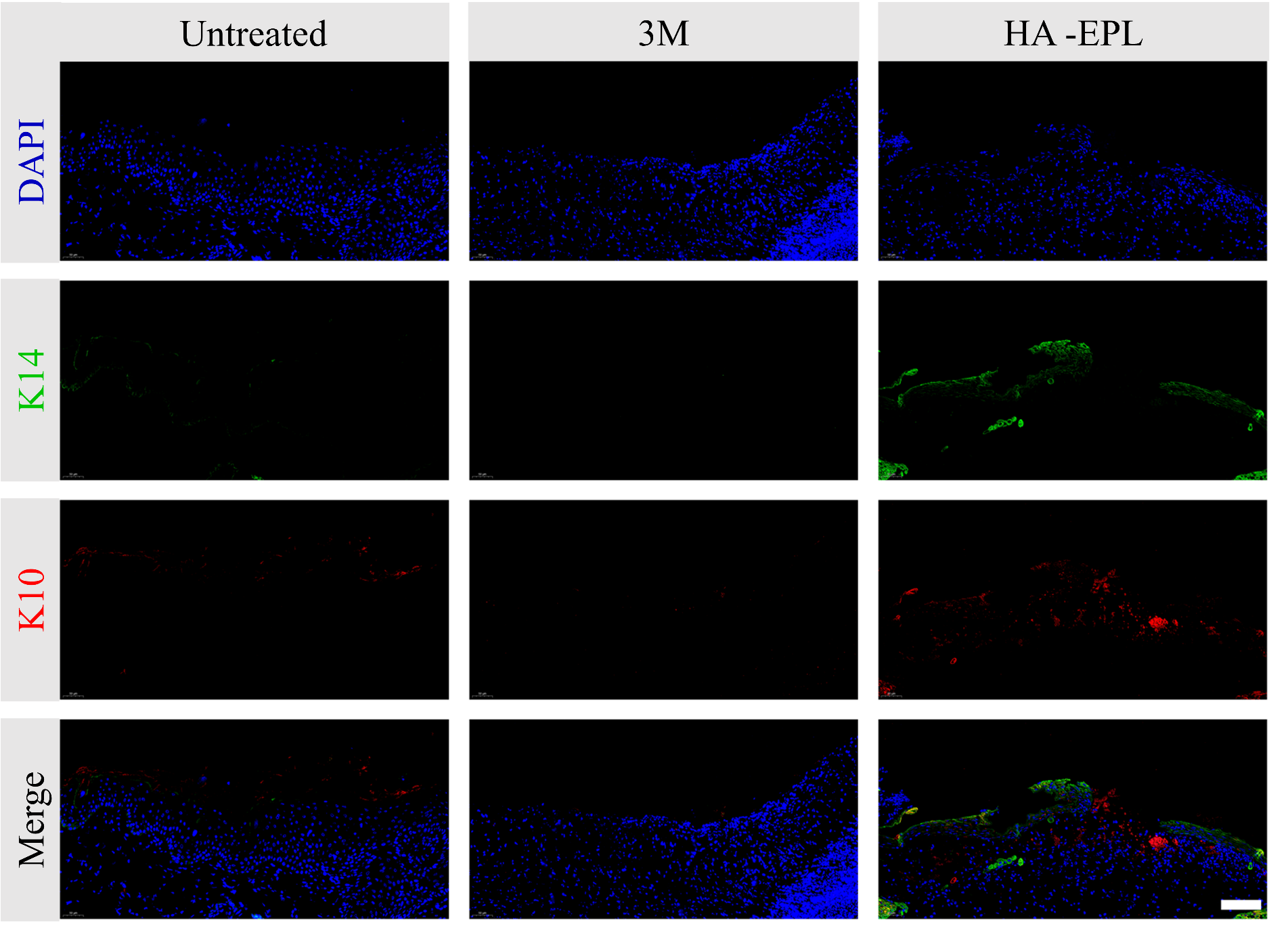


**Figure S16.** Double immunofluorescence staining of cytokeratin 10 (K10, red) and cytokeratin 14 (K14, green). Scale bar: 50 μm.


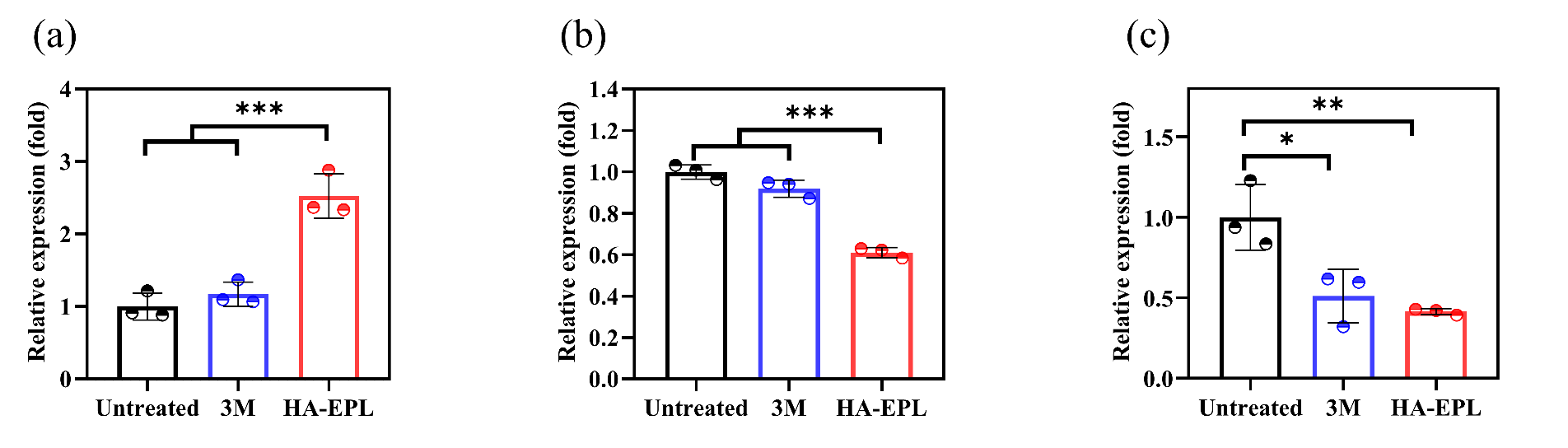


**Figure S17.** The gene expression around the full-thickness infected skin wound area was extracted and quantitively evaluated by q-PCR test (n = 3). (a) Relative expression of VEGF. (b) Relative expression of TNF-α. (c) Relative expression of IL-1β. **P* < 0.05, ***P* < 0.01, ****P* < 0.001.


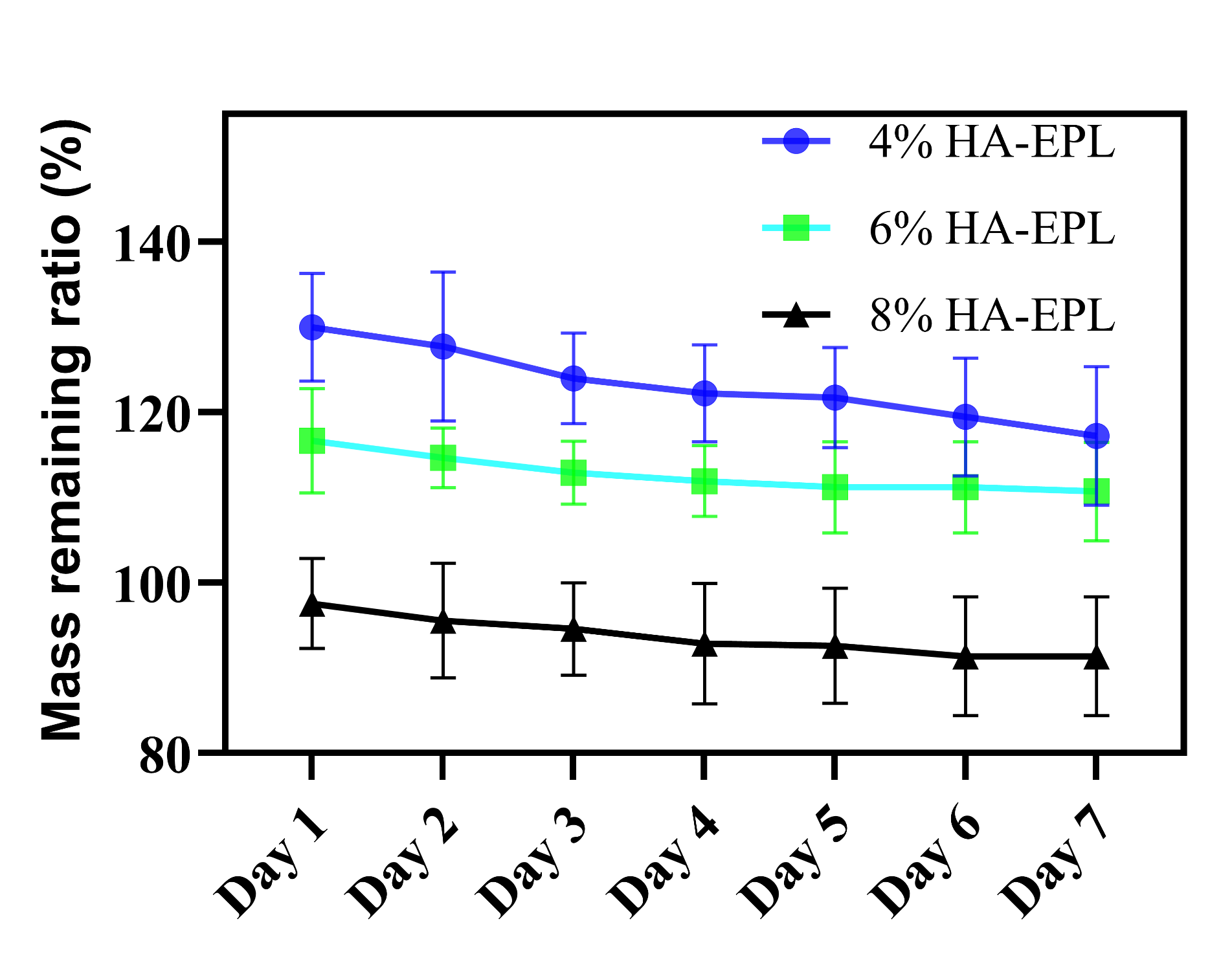


Figure S18. Degradation profile of HA-EPL coacervates in normal saline solution at 37 °C (n = 4).

**Reference**

1. Zhao, X., et al., Antibacterial anti-oxidant electroactive injectable hydrogel as self-healing wound dressing with hemostasis and adhesiveness for cutaneous wound healing. Biomaterials, 2017. 122: p. 34-47.

2. Chen, G., et al., Bioinspired Multifunctional Hybrid Hydrogel Promotes Wound Healing. Advanced Functional Materials, 2018. 28(33).

3. Shen, S., et al., An ultrasmall infinite coordination polymer nanomedicine-composited biomimetic hydrogel for programmed dressing-chemo-low level laser combination therapy of burn wounds. Chemical Engineering Journal, 2021. 426.

4. Liu, W., et al., Synthetic Polymeric Antibacterial Hydrogel for Methicillin-Resistant Staphylococcus aureus-Infected Wound Healing: Nanoantimicrobial Self-Assembly, Drug- and Cytokine-Free Strategy. ACS Nano, 2020. 14(10): p. 12905-12917.
